# RNA disruption is a widespread phenomenon associated with stress-induced cell death in tumour cells

**DOI:** 10.1038/s41598-023-28635-8

**Published:** 2023-01-31

**Authors:** Phillipe Butler, Isabella Pascheto, Michayla Lizzi, Renée St-Onge, Carita Lanner, Baoqing Guo, Twinkle Masilamani, Laura B. Pritzker, A. Thomas Kovala, Amadeo M. Parissenti

**Affiliations:** 1 Graduate Program in Chemical Sciences, Laurentian University, Sudbury, ON Canada; 2 Rna Diagnostics, Inc., Sudbury, ON Canada; 3 Rna Diagnostics, Inc., Toronto, ON Canada; 4 Health Sciences North Research Institute, Sudbury, ON Canada; 5 Division of Medical Sciences, Northern Ontario School of Medicine, Sudbury, ON Canada

**Keywords:** Cancer, Cell biology, Molecular biology, Biomarkers, Molecular medicine, Oncology

## Abstract

We have previously shown that neoadjuvant chemotherapy can induce the degradation of tumour ribosomal RNA (rRNA) in patients with advanced breast cancer, a phenomenon we termed “RNA disruption”. Extensive tumour RNA disruption during chemotherapy was associated with a post-treatment pathological complete response and improved disease-free survival. The RNA disruption assay (RDA), which quantifies this phenomenon, is now being evaluated for its clinical utility in a large multinational clinical trial. However, it remains unclear if RNA disruption (i) is manifested across many tumour and non-tumour cell types, (ii) can occur in response to cell stress, and (iii) is associated with tumour cell death. In this study, we show that RNA disruption is induced by several mechanistically distinct chemotherapy agents and report that this phenomenon is observed in response to oxidative stress, endoplasmic reticulum (ER) stress, protein translation inhibition and nutrient/growth factor limitation. We further show that RNA disruption is dose- and time-dependent, and occurs in both tumourigenic and non-tumourigenic cell types. Northern blotting experiments suggest that the rRNA fragments generated during RNA disruption stem (at least in part) from the 28S rRNA. Moreover, we demonstrate that RNA disruption is reproducibly associated with three robust biomarkers of cell death: strongly reduced cell numbers, lost cell replicative capacity, and the generation of cells with a subG1 DNA content. Thus, our findings indicate that RNA disruption is a widespread phenomenon exhibited in mammalian cells under stress, and that high RNA disruption is associated with the onset of cell death.

## Introduction

A recent analysis of pooled data in the National Cancer Database revealed that, of nearly 14,000 breast cancer patients undergoing neoadjuvant chemotherapy, 19% of patients achieved a pathological complete response (pCR), involving complete tumour destruction at the microscopic level^1^. In contrast, 17% of patients experienced no clinical response, and 20% of patients experienced disease progression^1^, highlighting the limited effectiveness of currently available chemotherapy regimens for the treatment of breast cancer. Moreover, the vast majority of breast cancer patients undergoing chemotherapy experience significant toxic side effects from the use of antineoplastic agents, including neutropenia, thrombocytopenia, vomiting/nausea, diarrhoea, stomatitis, mucositis, skin and subcutaneous tissue disorders, sensory neuropathy, hepatic toxicity, and cardiac disorders^2,3^. Thus, there is an urgent unmet need for reliable biomarkers of tumour cell death, both for consistently predicting patient response and outcome after neoadjuvant chemotherapy, and for in vitro anti-cancer drug discovery efforts. While the prediction of treatment response based on various prognostic and predictive biomarkers for breast cancer (such as stage and molecular subtype) has proven useful, the results are largely based on population-level analyses^4–6^. Ultimately, an individualized approach based on a particular patient’s response to treatment is advantageous for both higher treatment success rates and the reduction in treatment side effects.

For monitoring treatment response in breast cancer patients, only pCR (complete tumour destruction) is a sufficiently robust indicator of tumour cell death to be associated with improved survival after neoadjuvant chemotherapy^7^. However, pCR rates are scored post-treatment, preventing their use during treatment to identify patients with non-responding tumours for which treatment regimens should be modified or discontinued, both to increase the likelihood of treatment success and to minimize their associated side-effects. Metrics such as changes in tumour volume, tumour levels of proliferation markers such as Ki-67, or the apparent diffusion coefficient values for water in tumours can serve as biomarkers of chemotherapy treatment response, but their utility has been limited by the use of highly variable cut points in significantly underpowered studies^8,9^. Blood and serum markers such as CA15-3, CEA and HER2 have also been evaluated in this context, but these markers suffer from lack of specificity for breast cancer, and studies that used them were also significantly underpowered^10–13^. While the reduction of tumour ^18^F-deoxyglucose uptake (measured by positron emission tomography) following chemotherapy administration is associated with the achievement of pCR in breast cancer patients, the sensitivity and specificity of this approach have been limited, and the costs and infrastructure associated with positron emission tomography scanning are seen as major limiting factors^8,14^.

From a drug discovery perspective, most high-throughput biomarkers used to evaluate the efficacy of anti-cancer drugs (such as the 3-(4,5-dimethylthiazol-2-yl)-2,5-diphenyl tetrazolium bromide (MTT), dye exclusion, recovery and clonogenic assays) cannot distinguish between cytotoxic drug effects (induction of cell death) and cytostatic drug effects (arrest of tumour cell growth while cells remain viable). These assays all quantify decreases in cellular phenotypes associated with healthy growing cells (mitochondrial respiration, plasma membrane integrity, or replicative capacity) rather than cell death-related attributes^15–18^.

In 2010, we observed that chemotherapy treatment can strongly reduce tumour RNA integrity, inducing the formation of abnormal high-molecular-weight RNA species^19^. We later termed this phenomenon “RNA disruption”, due to the ability of chemotherapy treatment to disrupt the normal RNA banding pattern^20–22^. High RNA disruption in human breast cancer patients and canine lymphoma patients was found to be strongly associated with clinical response to treatment and improved disease-free survival after treatment^20–22^. Moreover, RNA disruption was shown to predict pCR as early as one cycle of chemotherapy in patients with HER2 + breast cancer^23,24^. Our most recent study has revealed that chemotherapy-induced RNA disruption in cultured A2780 ovarian tumour cells is associated with a reduction in cell numbers (cytotoxicity) and appears to be superior to the Cell Counting Kit-8 (CCK8), dye exclusion, recovery, and clonogenic assays at identifying cytotoxic drugs that promote cell destruction^25^. Nevertheless, several important questions relating to RNA disruption remain unanswered, including how widespread is the disruption phenomenon among cell types, how specific are the triggers for RNA disruption, and how tightly is it associated with cell death.

In this study, we explored whether RNA disruption is a common phenomenon among tumour cells undergoing cell death. We examined RNA disruption in four different tumour cell lines treated with a wide variety of chemotherapy drugs and, for the first time, with compounds activating specific cellular stress pathways. We also investigated whether RNA disruption can occur in non-tumourigenic cells, and whether statistically significant increases in RNA disruption are reproducibly associated with the onset of cell death. Finally, we examined whether the high-molecular-weight abnormal RNA bands generated during RNA disruption stem from the 28S rRNA.

## Results

### Doxorubicin-induced RNA disruption is both dose- and time-dependent in multiple tumour cell lines

**Figure 1:**
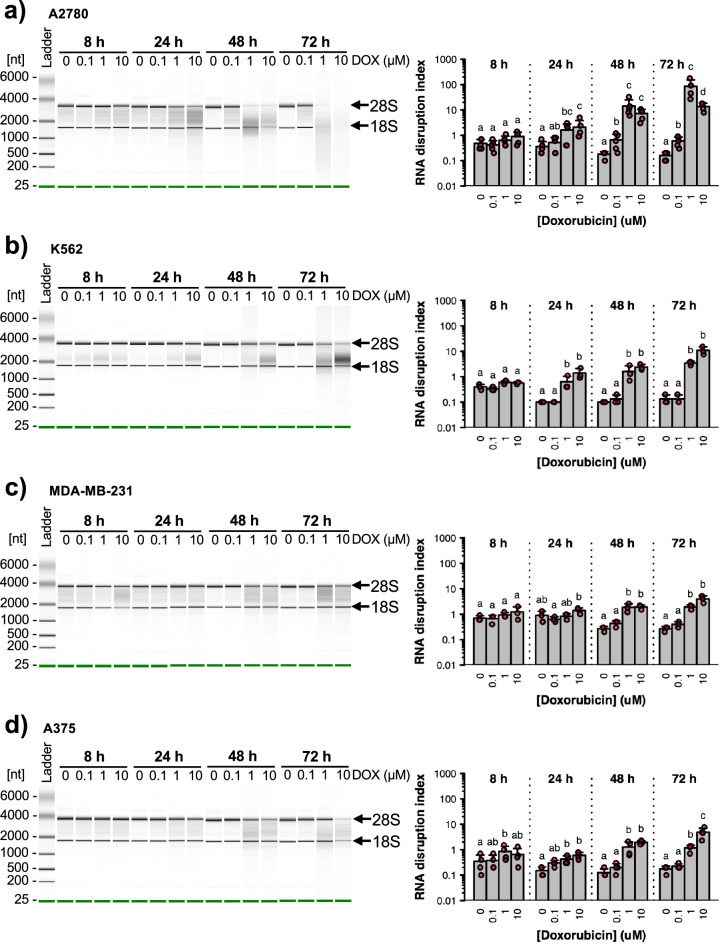
Dose- and time-dependent RNA disruption in response to doxorubicin. A2780 (**a**), K562 (**b**), MDA-MB-231 (**c**) and A375 (**d**) cells were exposed to different concentrations of doxorubicin (DOX) for 8 to 72 h. Total RNA was isolated from cells following drug treatment, and RNA disruption was analyzed using the RDA. *Left panels*. Virtual gel images of total RNA isolated from doxorubicin-treated cells. Arrows indicate the position of the full-length 28S and 18S rRNA bands. Each electropherogram is representative of at least three independent biological replicates. *Right panels*. RNA disruption quantified using the RDA. Data are presented as means ± standard deviation, with individual data points shown in red. A two-way ANOVA revealed a significant interaction between drug concentration and treatment time for all four cell lines [A2780, *F*(9, 62) = 18.27, *n* = 4–5, *P* < 0.01; K562, *F*(9, 32) = 7.68, *n* = 3, *P* < 0.01; MDA-MB-231, *F*(9, 32) = 8.75, *n* = 3, *P* < 0.01; A375, *F*(9, 48) = 6.22, *n* = 4, *P* < 0.01]. For a given treatment time, group pairs labelled with the same letter are not significantly different.

We began our enquiry by treating A2780 ovarian cancer cells with different concentrations of doxorubicin for varying lengths of time and measuring the amount of drug-induced RNA disruption using the RDA. With this assay, the extent of disruption is expressed using the RNA disruption index (RDI), a ratio between the sum of the areas of all abnormal peaks observed on RNA electropherograms and the sum of the areas of the 28S and 18S rRNA peaks. Except for dose–response curves, the drug concentration depicted in various figures represents the dose that produced maximum RNA disruption for the drug, while ensuring that sufficient RNA (> 5 ng µL^−1^) remained to obtain reliable electropherogram data and compute an RDI value. Doxorubicin induced strong RNA disruption in A2780 cells after at least 24 h, as seen by (i) a reduction in the intensity of the 28S and/or 18S rRNA bands and (ii) the appearance of RNA products in the “inter-region” (between the 28S and 18S rRNA bands), and the “fast-region” (below the 18S rRNA band) (Fig. 1a). In untreated cells, RNA disruption was minimal. Few RNA species were noted in the inter- and fast-regions, and low RDI values were calculated for the 0 µM doxorubicin doses (Fig. 1a). Similar results were obtained when treating cells with a low dose of doxorubicin (0.1 μM) for 8 or 24 h, though longer exposures did trigger mild RNA disruption (Fig. 1a). In contrast, higher doxorubicin doses (1 μM and 10 μM) significantly increased the levels of RNA disruption in A2780 cells after at least 24 h (Fig. 1a).

To determine if drug-induced RNA disruption is a common phenomenon in tumour cell lines, and not specific to the ovarian cancer cell line A2780, we treated three other tumour cell lines with doxorubicin. Similar to what was noted in drug-treated A2780 cells, 1 μM and 10 μM doxorubicin strongly induced RNA disruption in human K562 chronic myeloid leukemia cells (after 24 h) (Fig. 1b), human MDA-MB-231 breast carcinoma cells (after 48 h) (Fig. 1c) and human A375 melanoma cells (after 8–24 h) (Fig. 1d). However, 0.1 μM doxorubicin did not induce significant changes in RDI values for these latter three cell lines, irrespective of treatment duration (Fig. 1b–d).

The data depicted in Fig. 1 also illustrate that doxorubicin-induced RNA disruption is time-dependent. For all four investigated cell lines treated with a high doxorubicin dose (1 μM or 10 μM), RNA disruption increased in severity with time (Fig. 1). A2780 cells (but not K562, MDA-MB-231 and A375 cells) treated with a low dose of doxorubicin also displayed time-dependent RNA disruption (Fig. 1a). Untreated (0 μM) control cells did not exhibit abnormal bands on electropherograms, and RDI values were very low for all time points (Fig. 1). A two-way analysis of variance (ANOVA) revealed a significant interaction between doxorubicin concentration and exposure time for all cell lines (Fig. 1). RNA disruption was therefore found to be both dose- and time-dependent in all four cell lines investigated in this study.

### Multiple chemotherapy agents induce RNA disruption to varying extents

**Figure 2:**
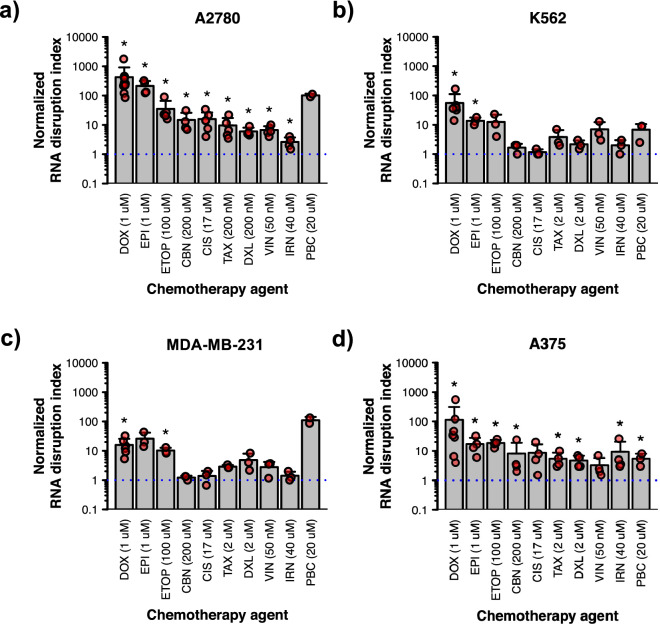
RNA disruption induced by multiple chemotherapy agents in different cell lines. A2780 **(a**), K562 (**b**), MDA-MB-231 (**c**) and A375 (**d**) cells were exposed to various chemotherapy agents for 72 h. Total RNA was isolated from cells following drug treatment, and RNA disruption was analyzed using the RDA. Data are presented as means ± standard deviation, with individual data points shown in red. Groups labelled with an asterisk possess a normalized RDI that is significantly greater than that of the untreated control (blue dotted line). See Supplementary Table S1 for detailed results of each statistical test. *DOX* doxorubicin, *EPI* epirubicin, *ETOP* etoposide, *CBN* carboplatin, *CIS* cisplatin, *TAX* paclitaxel, *DXL* docetaxel, *VIN* vincristine, *IRN* irinotecan, *PBC* palbociclib.

Having found that doxorubicin triggers RNA disruption in a variety of tumour cell lines, we next sought to determine whether other chemotherapy agents could also stimulate the disruption. To this end, we treated the above four tumour cell lines with multiple structurally and mechanistically distinct chemotherapy agents. RNA electropherograms are provided for these cell lines treated with various drugs at their optimal dose for inducing RNA disruption (Supplementary Fig. S1). For the A2780 cell line, RNA disruption was triggered most strongly by anthracyclines doxorubicin and epirubicin at their optimal doses, where there were very strong reductions in the intensities of the 28S and/or 18S rRNA bands and clear rRNA degradation products in the inter- and fast-regions (Fig. 2a, Supplementary Fig. S1). Several other chemotherapy agents also significantly stimulated RNA disruption in A2780 cells, including the topoisomerase II inhibitor etoposide, the platinating agents carboplatin and cisplatin, the taxanes paclitaxel and docetaxel, the vinca alkaloid vincristine and the topoisomerase I inhibitor irinotecan (Fig. 2a, Supplementary Fig. S1). The cyclin-dependent kinase 4/6 inhibitor palbociclib appeared to induce strong RNA disruption at its optimal dose based on visual inspection of RNA electropherograms (Supplementary Fig. S1); however, the increase in RDI was not statistically significant (Fig. 2a). The lack of statistical significance was likely owed to the small sample size and low statistical power. Isolation of sufficient RNA from palbociclib-treated cells was especially difficult, and so this resulted in few samples suitable for RDA analysis as particularly extensive disruption precludes successful RDI scoring.

Similar to the A2780 cell line, a variety of chemotherapy agents induced RNA disruption in three other tumour cell lines (Fig. 2, Supplementary Fig. S1). Statistically significant increases in RDI values were seen in A375 cells treated with any of the chemotherapy agents tested, with the exception of vincristine and cisplatin (Fig. 2d). RNA degradation products accumulated in K562 cells in response to most chemotherapy agents (Supplementary Fig. S1), but statistically significant increases in RDI values were only observed for doxorubicin and epirubicin (Fig. 2b). Similarly, higher levels of RNA degradation fragments were noted in MDA-MB-231 cells treated with a variety of chemotherapy agents at their optimal doses (Supplementary Fig. S1), but increases in RDI values were only statistically significant for doxorubicin and etoposide (Fig. 2c). Taken together, our data suggest that a wide variety of chemotherapy drugs can induce RNA disruption in various tumour cell lines originating from various tissues, although the magnitude of RNA disruption depends on the drug administered and the tumour cell line. Overall, the topoisomerase II inhibitors doxorubicin, epirubicin and etoposide were the most robust at inducing RNA disruption across the four tumour cell lines tested (Fig. 2, Supplementary Fig. S1).

### The activation of specific cellular stress pathways also triggers RNA disruption

**Figure 3:**
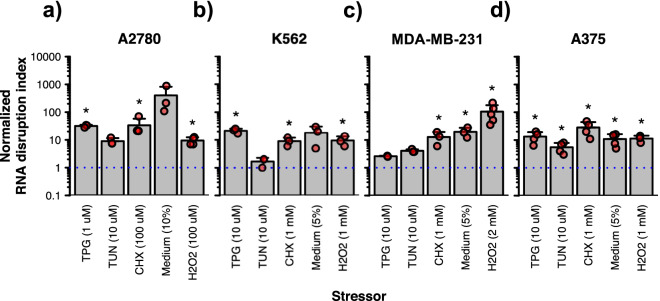
RNA disruption induced by various cellular stressors in multiple cell lines. A2780 (**a**), K562 (**b**), MDA-MB-231 (**c**) and A375 (**d**) cells were exposed to various cellular stressors for 72 h. Total RNA was isolated from cells following drug treatment, and RNA disruption was analyzed using the RDA. Data are presented as means ± standard deviation, with individual data points shown in red. Groups labelled with an asterisk possess a normalized RDI that is significantly greater than that of the untreated control (blue dotted line). See Supplementary Table S2 for detailed results of each statistical test. *TPG* thapsigargin, *TUN* tunicamycin, *CHX* cycloheximide, *Medium*, standard culture medium diluted to 5 or 10% in PBS.

We then investigated whether RNA disruption is only exhibited in tumour cells in response to anticancer agents, or whether other stressors can also induce RNA disruption in cells, including ER stress, protein translation inhibition, nutrient/growth factor limitation, and oxidative stress. In the A2780 cell line, the activation of ER stress pathways by either thapsigargin or tunicamycin (at their optimal doses) induced RNA disruption, as seen through reductions in the intensity of the 28S rRNA band and/or the increased presence of RNA degradation fragments on RNA electropherograms (Supplementary Fig. S2); however, statistically significant increases in RDI values were only seen for cells treated with thapsigargin (Fig. 3a). Similarly, whereas cycloheximide-induced inhibition of protein translation, nutrient/growth factor limitation resulting from culture medium dilution to 10%, and H_2_O_2_-induced oxidative stress, all promoted RNA disruption (as seen on RNA electropherograms) (Supplementary Fig. S2), statistically significant increases in RDI values were only seen for cycloheximide and H_2_O_2_ (Fig. 3a).

For the K562 cell line, while all tested stressors (except tunicamycin) induced the production of RNA degradation products (Supplementary Fig. S2), only thapsigargin, cycloheximide and H_2_O_2_ induced significant increases in RDI values (Fig. 3b). For the MDA-MB-231 and A375 cell lines, RDI values increased in response to each stressor, with the exception of thapsigargin and tunicamycin in the MDA-MB-231 cell line (Fig. 3c–d). Overall, like chemotherapy agents, the activation of several cellular stress pathways induced RNA disruption, although the magnitude of increases in RDI values varied depending upon the stressor and the tumour cell line tested.

### RNA disruption is associated with the onset of cell death

While our previous studies have shown that induction of RNA disruption by doxorubicin and cycloheximide is associated with cytotoxicity^25^, they did not assess whether RNA disruption is associated with the onset of cell death (as determined by meeting all three phenotypes commonly associated with cell death: reductions in cell numbers/cell destruction, cessation of cell replication, and the generation of cells with a subG1 DNA content). To begin this assessment, we examined a variety of chemotherapy agents for their effect on the number of cells in culture. The drugs were chosen based on their ability to induce high (doxorubicin) or low (docetaxel and irinotecan) RNA disruption in the A2780 cell line. A significant decrease in the number of cells in culture below that at the start of treatment would be indicative of cell destruction. However, it is worth noting that cell death or cell destruction can occur in a portion of the cell population without a net loss in cell numbers. Thus, we also employed other metrics for measuring cell viability and cell death. These included monitoring the ability of chemotherapy agents to inhibit or block cell proliferation post-treatment in the absence of drug (using recovery assays) or to generate cells with a non-viable subG1 DNA content (using flow cytometry after propidium iodide staining).

**Figure 4:**
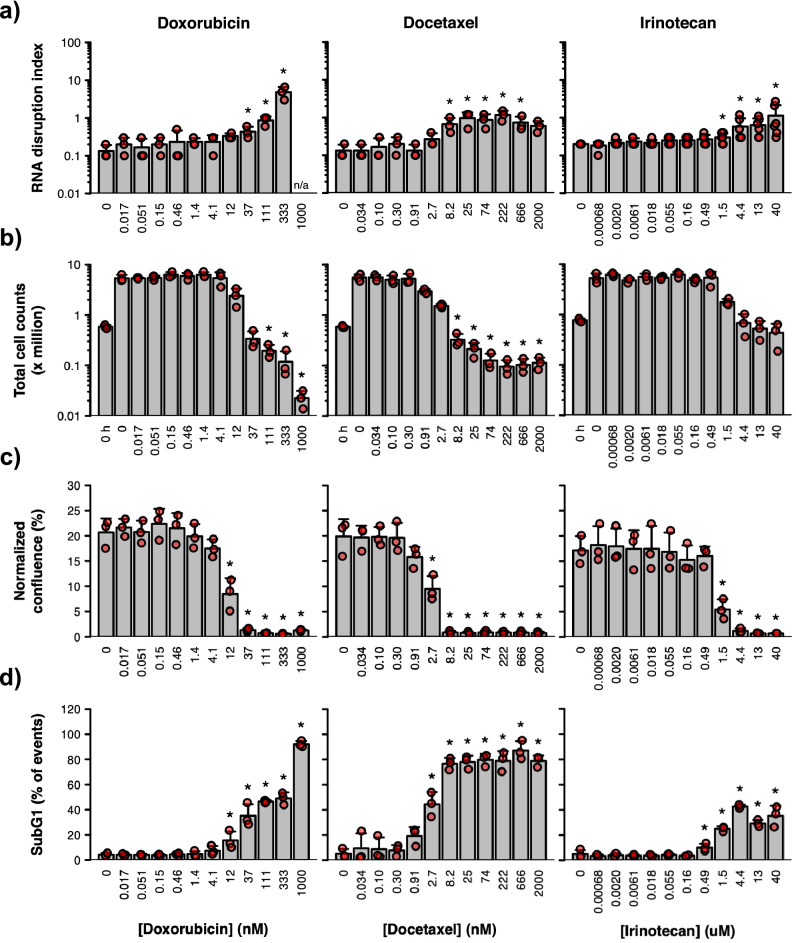
Effect of three chemotherapy agents on cell viability/death and RNA integrity. A2780 cells were treated with different concentrations of doxorubicin (left panels), docetaxel (middle panels) or irinotecan (right panels) for 72 h. (**a**) RNA disruption assay*.* Total RNA was isolated from cells, and RNA disruption was quantified using the RDA. (**b**) Cell counting assay. Total cells were counted using a haemocytometer following drug treatment. Untreated cells were counted prior to treatment (‘0 h’) to provide a baseline count. (**c**) Recovery assay. Drug-treated cells were collected, washed, resuspended in a drug-free medium and seeded into plates. After 96 h, the culture’s confluence was measured and normalized to its confluence at 2 h post-seeding. (**d**) DNA content analysis. Drug-treated cells were collected, washed, fixed, and stained with propidium iodide then analyzed by flow cytometry. Data are presented as means ± standard deviation, with individual data points shown in red. Groups labelled with an asterisk were significantly greater (panels a and d) or significantly lower (panels b and c) than the ‘0 h’ (panel b) or untreated (panels a, c and d) control. See Supplementary Table S3 for detailed results of each statistical test. n/a, extensive RNA disruption in the sample precluded RDA analysis.

For doxorubicin, RDI values were significantly increased when doxorubicin concentrations were equal to, or greater than, 37 nM (Fig. 4a). Cell counts revealed that doxorubicin doses at or above 111 nM were necessary to significantly reduce cell numbers below those at the start of treatment (Fig. 4b). However, cell replicative capacity in the absence of drug post-treatment was reduced at doxorubicin doses ≥ 12 nM (Fig. 4c), and a significant increase in the percentage of cells with a subG1 DNA content was obtained when doxorubicin concentrations were ≥ 12 nM (Fig. 4d, Supplementary Fig. S4). Taken together, these results indicate that RNA disruption, at the lowest dose where it occurs, is also accompanied by a loss of cell replicative capacity and the generation of non-viable cells with a subG1 DNA content.

While docetaxel did not induce strong RNA disruption (Supplementary Fig. S3), a significant increase in RDI values occurred at docetaxel concentrations ≥ 8.2 nM (Fig. 4a). At concentrations of docetaxel ≥ 8.2 nM, there were significant reductions in cell number (Fig. 4b), a complete loss of cell replication (Fig. 4c), and the vast majority of cells possessed a subG1 DNA content (Fig. 4d, Supplementary Fig. S4). Thus, docetaxel concentrations at or above those required to induce RNA disruption appeared to be associated with concentrations promoting cell death (as measured by three robust cell death biomarkers).

Irinotecan did not induce strong cell death compared to both doxorubicin and docetaxel in vitro. No statistically significant reductions in cell numbers were observed for irinotecan even at the highest doses (Fig. 4b), and the four highest doses tested caused only 25–45% of cells or cell fragments to acquire a subG1 DNA content (Fig. 4d, Supplementary Fig. S4). This is in contrast to doxorubicin, which dramatically reduced cell numbers by 96% (Fig. 4b), and caused almost 100% of cells to acquire a subG1 DNA content (Fig. 4d, Supplementary Fig. S4). Docetaxel also induced cell death to a higher extent than irinotecan with a reduction in cell numbers by 81% (Fig. 4b) and the accumulation of subG1 DNA in 80% of cells at the highest dose (Fig. 4d, Supplementary Fig. S4). The lower ability of irinotecan to induce RNA disruption and cell death (by three metrics) in vitro may be due to its properties as a pro-drug. Irinotecan needs to be converted by liver carboxylesterases into its active metabolite (SN-38) in humans; SN-38 is 1000 times more active than irinotecan^26^. Nevertheless, it is interesting that doses of irinotecan (≥ 1.5 µM) that induced a statistically significant elevation in RDI (Fig. 4a) also suppressed cellular proliferation (Fig. 4c) and promoted an increase in the percentage of cells with a subG1 DNA content (Fig. 4d, Supplementary Fig. S4). Taken together, our data indicate that significant increases in RNA disruption are associated with changes in three phenotypes associated with cell death: cessation of cell proliferation, reduced cell numbers (cell destruction), and the generation of cells with a non-viable subG1 DNA content. This may help explain the RDA’s utility at predicting complete tumour destruction and improved survival after neoadjuvant chemotherapy in breast cancer patients^21^.

### RNA disruption is also observed in some non-tumourigenic cell types

**Figure 5:**
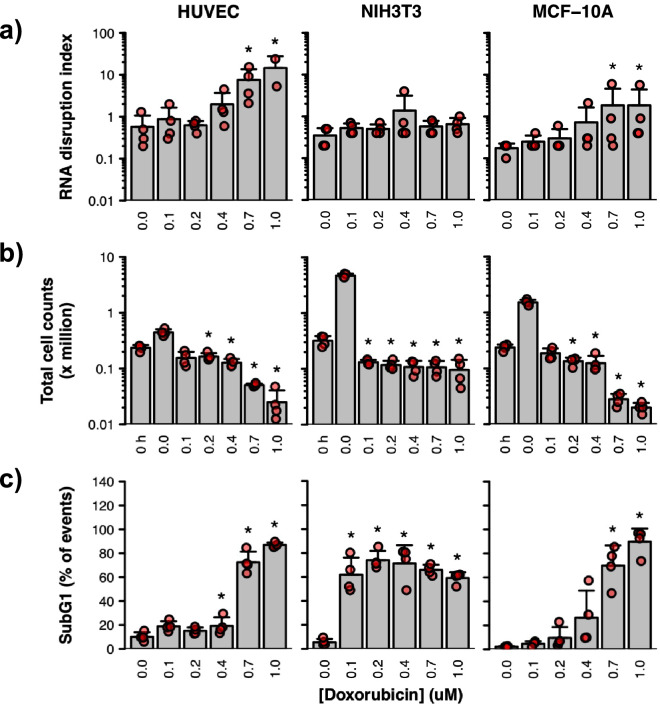
Effect of doxorubicin on cell viability/death and RNA integrity in three non-tumourigenic cell lines. Human umbilical vein endothelial cells (HUVECs) (left panels), mouse NIH3T3 fibroblast cells (middle panels) and human MCF-10A breast epithelial cells (right panels) were treated with doxorubicin for 72 h. (**a**) RNA disruption assay. Total RNA was isolated from cells, and RNA disruption was quantified using the RDA. (**b**) Cell counting assay. Total cells were counted using a haemocytometer following drug treatment. Untreated cells were counted prior to treatment (‘0 h’) to provide a baseline count. (**c**) DNA content analysis. Drug-treated cells were collected, washed, fixed and stained with propidium iodide then analysed by flow cytometry. Data are presented as means ± standard deviation, with individual data points shown in red. Groups labelled with an asterisk were significantly greater (panels a and c) or significantly lower (panel b) than the ‘0 h’ (panel b) or untreated (panels a and c) control. See Supplementary Table S4 for detailed results of each statistical test.

We then examined whether RNA disruption could be manifested in several non-tumourigenic cell lines, including human umbilical vein endothelial cells (HUVECs), mouse NIH3T3 fibroblast cells, and human MCF-10A breast epithelial cells. As shown in Fig. 5a, statistically significant increases in RDI values were observed in MCF-10A cells and in HUVECs when treated with doxorubicin. Interestingly, the dose-dependent increases in the RDI nicely paralleled the dose-dependent reductions in cell number (Fig. 5b) and dose-dependent increases in the proportion of cells with a subG1 DNA content (Fig. 5c, Supplementary Fig. S5). In contrast, doxorubicin was unable to induce statistically significant increases in RDI values for NIH3T3 cells (Fig. 5a), although significant decreases in cell numbers were observed (Fig. 5b), along with strong increases in the percentage of these cells exhibiting a subG1 DNA content (Fig. 5c, Supplementary Fig. S5).

### RNA disruption products in the inter-region of RNA electropherograms stem largely from the 28S rRNA

**Figure 6:**
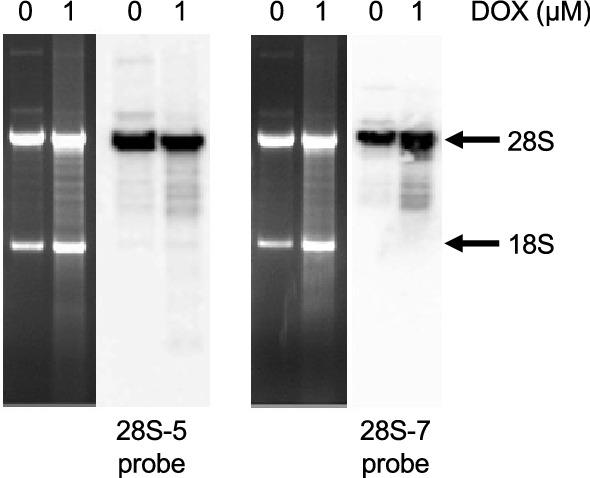
Origin of RNA disruption products. Total RNA was isolated from A2780 cells treated with doxorubicin (DOX) for 48 h. The RNA was then resolved by denaturing gel electrophoresis and transferred onto a PVDF membrane prior to hybridization with radiolabeled DNA probes specific to the 28S rRNA. UV-visualized, ethidium bromide-stained agarose gel images (left panels) and autoradiograms of the northern blots (right panels) are shown for the respective 28S rRNA probes. Arrows indicate the position of the full-length 28S and 18S rRNA bands. Each gel image and autoradiogram are representative of two independent biological replicates. Gel images and autoradiograms were cropped for clarity and conciseness. Full-length, uncropped gel images and autoradiograms are available in Supplementary Fig. S7.

The disappearance of the 28S rRNA and/or the appearance of a diffuse set of abnormal RNA species in chemotherapy-treated cells (Fig. 1, Supplementary Figs. S1 and S3) suggest that chemotherapy agents promote the degradation of the 28S rRNA in tumour cells into a broad range of high-molecular-weight fragments that appear in the inter-region of RNA electropherograms, between the 28S and 18S rRNA bands. To test this hypothesis, we conducted northern blotting experiments using total RNA isolated from doxorubicin-treated A2780 cells and radiolabelled probes that bind the 5′ or 3′ ends of the 28S rRNA. As shown in Fig. 6 and Supplementary Fig. S7, total RNA isolated from untreated cells (0 µM doxorubicin) exhibited very strong 28S and 18S rRNA bands, as well as two higher-molecular-weight RNA bands that likely represent the unprocessed (47S/45S) primary rRNA transcript and a partially processed 32S rRNA transcript, both of which contain the 28S rRNA sequence^27^. Upon treatment of cells with 1 μM doxorubicin for 48 h, the isolated total RNA, when viewed on ethidium bromide-stained gels, showed elevated levels of a number of RNA bands in the inter- and fast-regions (Fig. 6, Supplementary Fig. S7). When the same RNA preparations were probed for 28S rRNA-specific transcripts, we found that a number of these high-molecular-weight inter-region RNA bands bound both 28S rRNA probes (Fig. 6, Supplementary Fig. S7), supporting the hypothesis that these bands are 28S rRNA degradation products generated in cells upon doxorubicin treatment. Interestingly, the two transcripts of molecular weight greater than that of the 28S rRNA (described above) were also seen on the northern blots, and their intensities appeared to decrease upon doxorubicin treatment (Fig. 6, Supplementary Fig. S7), suggesting that they are also degraded during RNA disruption and may contribute to the doxorubicin-induced RNA degradation products observed in the inter- and fast-regions of electropherograms from treated cells. Interestingly, very low levels of RNA degradation products in the inter-region were present in untreated cells (Fig. 6, Supplementary Fig. S7), suggesting that these degradation products can form without doxorubicin treatment, possibly through a normal cellular process for downregulating rRNAs.

## Discussion

### Inducers of rRNA degradation (RNA disruption) and their associated cellular pathways

As illustrated in the current study, we found that RNA disruption is a widespread, dose- and time-dependent phenomenon observed in response to a variety of structurally and mechanistically distinct chemotherapy agents in cell lines derived from tumours of various cell types, including breast epithelial cells, ovarian epithelial cells, myeloid cells, and melanocytes (Figs. 1, 2). RNA disruption was also observed in non-tumourigenic cell lines (Fig. 5), further underscoring the widespread nature of this observed phenomenon. This study extends and supports our prior work in breast and ovarian tumour cells^28^. The wide variety of high-molecular-weight rRNA degradation fragments induced by chemotherapy agents in our in vitro studies (Fig. 1, Supplementary Figs. S1 and S3) closely resembles that found in tumour biopsies from breast cancer patients^21^. Our study also provides evidence that the diffuse bands of high-molecular-weight RNA fragments in the inter-region of RNA electropherograms generated during chemotherapy-dependent RNA disruption stem (at least in part) from the 28S rRNA (Fig. 6, Supplementary Fig. S7). It also reveals that RNA disruption can be induced in cells following exposure to specific cell stressors, including ER stress, oxidative stress, and nutrient/growth factor limitation (Fig. 3, Supplementary Fig. S2). Finally, we provide evidence of a strong relationship between the induction of RNA disruption and cell death, as measured by three criteria: reduction in cell numbers, loss of cell replicative capacity, and generation of cells with a subG1 DNA content (Figs. 4,5).

The phenomenon of stress-induced rRNA degradation was first reported by Houge et al*.* in 1993^29^ and was later observed in various in vitro models in response to numerous chemical agents and cell stressors, including cAMP analogs^29,30^, okadaic acid^30^, tumour necrosis factor with cycloheximide^30^, victorin^31^, acetic acid^32^, H_2_O_2_^32^, ER stressors^33^, cell aging^32^, glucose and sorbitol starvation^32^, nitrogen starvation^34^, anisomycin^35^, satratoxin G^35^, ricin^35^ and various recombinant vaccinia viruses^36,37^. However, these previous studies did not use metrics such as the RDI to quantify the magnitude of rRNA degradation in response to specific agents in order to reliably discern possible differences in their abilities to induce rRNA degradation. Strictly speaking, all abnormal bands on RNA electropherograms need not stem from the degradation of 28S and/or 18S rRNAs. Some abnormal bands may be due to changes in the levels of other abundant RNA transcripts. Nevertheless, based on northern blotting experiments performed in the current study (Fig. 6, Supplementary Fig. S7), at least some of the abnormal bands in the inter-region of RNA electropherograms stem from the 28S rRNA. We thus refer to this phenomenon (measured by quantifying peaks on RNA electropherograms) as “RNA disruption”, where chemotherapy agents and cellular stressors have the capacity to disrupt the normal total RNA banding pattern.

Extending our previously published findings^28^, we have observed in the current study that the extent of RNA disruption depended upon the specific chemotherapy agent administered. These different levels of RNA disruption may reflect the relative sensitivity of the cell lines to specific chemotherapy agents and stressors. Consistent with this view, we have shown a clear association between the sensitivity of specific cell lines to known chemotherapy agents (as measured using clonogenic assays) and the ability of such agents to induce RNA disruption^25,28^. Additional factors likely play a role in the degree of RNA disruption induced by specific chemotherapy agents or stressors, including the drug’s mechanism of action and its eventual effects on ribosomes. For example, the anthracyclines doxorubicin and epirubicin, which inhibit topoisomerase II and induce DNA damage, consistently induced stronger RNA disruption than the microtubule-stabilizing agents docetaxel and paclitaxel (Fig. 2, Supplementary Fig. S1).

It is interesting that the four chemotherapy agents that most strongly induced RNA disruption are the topoisomerase II inhibitors doxorubicin, epirubicin, and etoposide, and the cdk4/6 inhibitor palbociclib (Fig. 2, Supplementary Fig. S1). There may be a mechanistic link between the inhibition of topoisomerase II and/or the blocking of cell cycle progression and ribosome turnover mechanisms such as the non-functional rRNA decay (NRD) pathway^38^. This pathway identifies mutations within key regions of the 28S and 18S rRNAs that render the rRNAs non-functional, and subsequently targets the non-functional RNAs for degradation. Another possible mechanism for RNA disruption may involve stress-induced activation of pathways promoting selective autophagic degradation of mature ribosomes, a process known as ribophagy^39^. Interestingly, the cleavage of ubiquitinated ribosomal proteins occurs in both 28S/18S NRD and ribophagy, and may play a role in exposing both rRNAs to degradation by specific RNases associated with these processes^40,41^. An additional mechanism that may contribute to RNA disruption involves the chemical, non-enzymatic cleavage of rRNA. Zinskie and colleagues^42^ recently demonstrated that yeast cells lacking the mitochondrial glutathione-dependent oxidoreductase gene *grx5* exhibit stress-induced breaks in the 25S rRNA that correlate with increasing intracellular iron levels and appear to be due to a Fenton reaction-induced hydroxyl radical production. However, the rRNA degradation fragments generated by the iron-mediated 25S rRNA strand breaks are of very defined molecular weights, unlike the diffuse pattern of rRNA fragments we have seen in RNA disruption^42^. The anthracyclines doxorubicin and epirubicin, besides being topoisomerase II inhibitors, are strong inducers of reactive oxygen species (ROS)^43^. In contrast, the taxanes generate some, but considerably less, ROS^43^. If ROS generation by chemotherapy agents plays a role in RNA disruption, then this could explain why the anthracyclines promote much more RNA disruption than the taxanes (Fig. 2, Supplementary Fig. S1). It should be noted, however, that a recent study by Yokoyama and colleagues suggests that both doxorubicin and the taxane paclitaxel generate equivalent amounts of ROS using the CellROX assay^43^. Moreover, whereas etoposide does not appear to induce significant levels of ROS in the CellROX assay^43^, the drug was able to induce strong RNA disruption in our study. The reason for these contrasting observations may be related to differences in the drug doses or cell lines employed in the various studies. Consistent with a role for ROS generation in RNA disruption, we observed in this study that the strong ROS-generator H_2_O_2_ was able to reproducibly induce RNA disruption in all four tumour cell lines tested (Fig. 3, Supplementary Fig. S2); moreover, H_2_O_2_-induced RNA disruption occurred in a much shorter time period than RNA disruption triggered by chemotherapy agents (unpublished findings). ROS have the capacity to induce a variety of mutations in RNA including guanine base oxidation, strand scission, and rRNA-protein cross-links (reviewed in Gilles et al*.*^44^) that could result in the generation of rRNA mutations known to activate the 28S/18S NRD pathways. Moreover, ROS-mediated RNA strand breaks are also known to promote ribophagy^45,46^. As stated above, ROS can also facilitate the creation of non-enzymatic rRNA strand breaks through a Fenton reaction involving hydroxyl radical production^42^. Thus, ROS production by chemotherapy agents and cellular stressors could facilitate RNA disruption through a variety of chemical reactions and enzymatic processes that occur in cells. Interestingly, cancer cells generate much higher levels of ROS than non-tumour cells, and higher cellular levels of ROS are associated with malignant transformation of cells^47^. Thus, tumour cells, in particular, may be predisposed to stress-induced RNA disruption. However, we do show in this study that doxorubicin-induced RNA disruption can be observed in non-tumourigenic MCF-10A cells and HUVECs (Fig. 5a), indicating that RNA disruption is not unique to tumour cells, but could represent a stress-induced pathway of ribosomal turnover operating in many cell types. The possible association of ROS generation with RNA disruption may also help explain why chemotherapy-dependent RNA disruption is both time- and dose-dependent.

In this study, we evaluated the association between RNA disruption and cell death using multiple measures of cell viability. This helps provide biological relevance for increases in RNA disruption in cells. Mapletoft et al*.*^25^ demonstrated in an earlier study that the RDA is a valuable tool for the discovery of drugs that promote tumour cell death. It is much less affected by other changes in cellular phenotypes associated with drug cytotoxicity, including reductions in cellular replication, mitochondrial metabolism, or plasma membrane integrity^25^. In our current study, we provide strong evidence that statistically significant increases in RDI values are consistently associated with three robust biomarkers associated with cell death and destruction. These include a reduction in cell numbers below their pre-treatment values, loss of cell replicative capacity, and increases in the percentage of cells with a non-viable subG1 DNA content. Our data therefore suggest that stress-induced RNA disruption is reproducibly associated with cell destruction and the onset of cell death by several stressors and a variety of structurally and mechanistically distinct chemotherapy agents. Since all of the tested stressors and chemotherapy drugs in our study induced both cell death and RNA disruption, it can be hypothesised that RNA disruption is a common event that occurs downstream of many cell death pathways.

Interestingly, a variety of pathways associated with cell death can be activated by the generation of ROS. For example, ROS can oxidize permeability transition pore channels within mitochondria, resulting in the release of a variety of pro-apoptotic factors^48^. In addition, protein oxidation within the ER can induce the unfolded protein response^49^, which activates pro-apoptotic pathways when left unchecked^50^. High ROS levels also promote autophagy^51^, which results in autophagic cell death when sustained^52,53^. Moreover, the strong RNA disruption agent doxorubicin is known to promote autophagy in various tumour cell lines, including the A2780 and MDA-MB-231 cell lines used in this study^54,55^. Ribophagy has also been associated with cell death by ROS-promoting agents^56^, although most studies have involved starvation of cells rather than their treatment with ROS-inducing agents^41^. Interestingly, limiting nutrient and growth factor availability by diluting the cell culture medium with phosphate-buffered saline solution (PBS), which would be expected to induce autophagy, also triggered extensive RNA disruption in our current study (Fig. 3, Supplementary Fig. S2). Transient activation of NRD and ribophagy pathways may permit cells to survive exposure to cellular stressors and chemotherapy agents by reducing the energetically costly process of protein translation. However, prolonged activation of these pathways would be expected to promote cell death through organelle destruction and the activation of cell death pathways.

### Association of RNA disruption with tumour cell death

Data presented in this manuscript and previously published evidence^25^ depict a strong association between RNA disruption and cell death. For example, in a previous study, we showed that RNA disruption occurred in cycloheximide-treated A2780 cells only when drug concentrations were sufficiently high to trigger cell death. Drug concentrations known to promote cell cycle arrest, but not cell death, did not promote RNA disruption. Consistent with these earlier findings, we show here that the dose required by doxorubicin to induce RNA disruption in tumourigenic A2780 cells (Fig. 4) and non-tumourigenic MCF-10A cells and HUVECs (Fig. 5) is similar to that required to induce cell death in these cells, as measured by DNA content analysis (Figs. 4, 5). Coupled with the association of on-treatment tumour RNA disruption with a post-treatment pCR^19,21,23^, our studies strongly support the hypothesis that RNA disruption is robustly associated with cell death in vitro and in vivo. In further support of this hypothesis, we recently reported in abstract form that RNA disruption was found to be associated with tumour cell death induced by exogenously added immune cells^57^. We observed that natural killer cells from human volunteers could induce both RNA disruption and cell death in K562 chronic myelogenous leukemia cells. A manuscript documenting these findings has been completed for publication.

Given the strong relationship between RNA disruption and cell death outlined above, the RDA is particularly apt at detecting genuine changes in cell viability. This is in contrast to many classical drug sensitivity assays which are unable to discriminate between cell cycle arrest and cell death, and whose results can be misinterpreted to mean a loss in viability^25^. Although subG1 DNA content analysis of tumour cells also offers an accurate means of quantifying cell death, this approach is not feasible when working with tumours from humans or animals, as cells within tumours are held together through adhesion to each other and to the extracellular matrix. This makes flow cytometric assessment of DNA content in tumours of cancer patients untenable. In contrast, the RDA is not limited to investigations involving disaggregated cells, as evidenced by our previous works with tumour biopsies from human breast cancer patients^21,23^ and canine lymphoma patients^22^.

We observed a similar association of RNA disruption with cell death for non-tumourigenic cell lines, except for NIH3T3 fibroblast cells, where no statistically significant increase in RNA disruption was observed in the presence of doxorubicin, despite the ability of the drug to induce significant reductions in cell numbers and strong increases in the percentage of cells with a subG1 DNA content (Fig. 5, Supplementary Fig. S5). The reason for this exception for NIH3T3 fibroblasts is unclear. One possible explanation for this may be that these cells are particularly sensitive to doxorubicin, as evidenced by the generation of large numbers of cells with a subG1 DNA content at a much lower concentration of doxorubicin (0.1 μM) (after 72 h of treatment) compared to HUVECs and MCF-10A cells (Fig. 5, Supplementary Fig. S5). Moreover, substantial increases in NIH3T3 cells with a subG1 DNA content could be seen after only 24 h of treatment with 0.4 μM, 0.7 μM, or 1.0 μM doxorubicin (Supplementary Fig. S6). These observations suggest that NIH3T3 cells die so rapidly and in such large numbers in response to doxorubicin that the time-dependent process of RNA disruption does not occur.

### Potentiation of doxorubicin-induced RNA disruption in mammalian cells by infection with Mycoplasma

The broad diffuse bands of rRNA degradation fragments induced in cultured tumour cells by chemotherapy agents and cell stressors (residing in the inter- and fast-regions of electropherograms) are very different from the more limited number of discrete rRNA degradation fragments seen in our first in vitro study documenting this phenomenon^28^. The latter banding pattern was found to be due to *Mycoplasma* contamination of the cell lines used (Supplementary Fig. S8). A2780 cells, with no prior history of *Mycoplasma* infection, and *Mycoplasma-*infected A2780 cells treated with an antibiotic capable of killing *Mycoplasma*, consistently exhibited the diffuse pattern of rRNA degradation products (Supplementary Fig. S8). Further confirming the effect of *Mycoplasma* infection on the rRNA degradation pattern, re-infection of A2780 cells with *Mycoplasma* restored the previously observed pattern of discrete RNA disruption products (Supplementary Fig. S8). The ability of RNA disruption to occur in *Mycoplasma*-infected cells, albeit in a different form, attests to the importance of this pathway in cellular stress response.

### On-treatment tumour RNA disruption as a clinically valuable biomarker to predict patient outcome after neoadjuvant chemotherapy

Many cancer patients undergoing neoadjuvant chemotherapy suffer from the side effects of chemotherapy exposure, while drawing no survival benefit from therapy^1–3,58^. Consequently, there is an unmet need for a chemotherapy response tool that can accurately distinguish between chemo-responsive and chemo-resistant tumours early in treatment. This tool would enable patients to forgo the costs and toxic side effects of ineffective chemotherapy regimens and enable non-responding patients to proceed more rapidly to alternate treatments.

At first glance, a biomarker associated with cell death from a broad variety of stimuli in both cancer and non-cancer cells might seem to be of little value, as its lack of specificity may hinder its ability to definitively identify a particular pathology or to quantify cell death in tumours induced by a particular anti-cancer agent with a specific mechanism of action. One might think that this would limit its translational value. However, it is important to note that tumour cells in cancer patients die by a number of different mechanisms in response to a wide variety of stimuli. For example, radiation induces tumour cell death in cancer patients by promoting mitotic catastrophe, apoptosis (including necroptosis and ferroptosis), necrosis, autophagy and immunogenic cell death. This depends on several factors, including tumour cell genetics, radiation dose and schedule, and the tumour microenvironment within the host^59^. Anti-cancer drugs can induce tumour cell death in cancer patients by these same varied mechanisms, depending upon identical factors^60,61^. Thus, a biomarker that could reliably identify dying tumour cells (regardless of the tissue of origin, the death-inducing agent or patient factors) would be extremely valuable, particularly if the biomarker can effectively predict treatment outcome and survival in patients. Consistent with this view, high tumour RNA disruption during neoadjuvant chemotherapy using various regimens has been shown in four published studies to predict patient outcome (clinical or pathologic complete response) and/or survival after treatment^21–23,62^. This strong association between tumour RNA disruption and patient outcome after neoadjuvant chemotherapy is now being further assessed in an ongoing international clinical trial called BREVITY (https://clinicaltrials.gov/ct2/show/NCT0352443). Because RNA disruption has been shown in the current manuscript to be time-dependent, the extent of tumour RNA disruption (RDI cut points) and other assay parameters will need to be pre-specified. These parameters include: (a) the number of days after the initiation of chemotherapy that biopsies are taken, (b) sample preservation, transport, and handling procedures, and (c) treatment regimens assessable by the RDA.

In summary, this paper describes the widespread phenomenon of RNA disruption (stress-induced rRNA degradation), which occurs in both tumourigenic and non-tumourigenic cell lines upon exposure to a variety of mechanistically distinct chemotherapy agents and cellular stressors. RNA disruption is both time- and dose-dependent, and the high-molecular-weight rRNA degradation products appear to stem (at least in part) from the 28S rRNA. The appearance of RNA disruption products (as measured using the RDI) is strongly associated with several biomarkers of cell death, including reductions in cell numbers below pre-treatment values, a suppression of cell replication, and the increased formation of cells with a subG1 DNA content. Future studies will focus on investigating the biochemical pathways by which chemotherapy agents and cellular stressors induce RNA disruption, including the production of ROS, the ubiquitination of specific ribosomal proteins, ribosomal protein proteolysis, the activation of known rRNA degradation pathways (such as ribophagy and the 28S and 18S NRD pathways), and the relationship between these various pathways and the activation of specific cell death mechanisms.

## Methods

### Ethics approval

This study did not require ethics approval from an ethics review committee because the study did not involve animals, humans, human data or material collected directly from animals or humans.

### Cell culture

The A2780 (ovarian endometrioid carcinoma) cell line^56^ was purchased from the European Collection of Authenticated Cell Cultures and was maintained in HyClone RPMI-1640 culture medium with L-glutamine and without HEPES (Thermo Fisher Scientific). The K562 (chronic myelogenous leukemia), MDA-MB-231 (breast adenocarcinoma), A375 (malignant melanoma), MCF-10A (breast epithelial) and NIH3T3 (mouse embryo fibroblast) cell lines were purchased from the American Type Culture Collection and were maintained in HyClone IMDM (K562; Thermo Fisher Scientific), Invitrogen DMEM (MDA-MB-231, NIH3T3 and A375; Life Technologies) and Lonza MEGM (MCF-10A; Cedarlane) cell culture medium. The Lonza MEGM medium was supplemented with the components of the Lonza MEGM BulletKit (Cedarlane), 100 ng mL^−1^ cholera toxin (Sigma) and 5% horse serum (Thermo Fisher Scientific). HUVECs were obtained from VEC Technologies and cultured in RPMI 1640 medium supplemented with 20% Cytiva HyClone fetal bovine serum (FBS; Thermo Fisher Scientific), 0.1 mg mL^−1^ heparin (Sigma) and 0.3 mg mL^−1^ endothelial cell growth supplement (EMD Millipore). All culture media were supplemented with 10% Cytiva HyClone FBS, unless otherwise specified. Cell cultures were maintained in Corning 75-cm^2^ (A2780,MDA-MB-231, NIH3T3, HUVEC, and MCF-10A) or Sarstedt 75-cm^2^ (A375 and K562) flasks for adherent (A2780, MDA-MB-231 A375, HUVEC, NIH3T3, and MCF-10A) or suspension (K562) cells. All cultures were maintained in a humidified incubator at 37ºC with 5% CO_2_ and passaged every 2–3 days using 0.25% trypsin–EDTA (Gibco), with the exception of the non-adherent K562 cell line, which did not require trypsin–EDTA for passage. Unless otherwise stated, all cell lines were confirmed to be free of *Mycoplasma* using a PCR-based *Mycoplasma* detection kit from Applied Biological Materials.

### Treatment of cultures with chemotherapy drugs and chemical stressors

Chemotherapy drugs (doxorubicin, epirubicin, etoposide, carboplatin, cisplatin, paclitaxel, docetaxel, vincristine and irinotecan) were donated by the pharmacy at Health Sciences North (Sudbury, ON, Canada). Palbociclib isethionate (PD-0332991) was purchased from MedChemExpress. Thapsigargin, tunicamycin, cycloheximide and hydrogen peroxide were purchased from Sigma-Aldrich. Nutrient- and growth factor-poor culture medium comprised FBS-supplemented culture medium diluted 1:10 or 1:20 with PBS, without Mg^2+^ and Ca^2+^.

Adherent cells were seeded in a 6-well Sarstedt tissue culture plate at a density of 250,000 cells per well in 3 mL drug-free medium. After 24 h, the culture medium was replaced with 3 mL fresh drug-free medium (untreated) or fresh medium containing chemotherapy drugs or chemical stressors. Suspension cells were plated and treated simultaneously.

### RNA disruption assay

RNA integrity was assessed using the RDA as described in our prior study^21^. Briefly, total RNA was isolated from drug- and stressor-treated cells, resolved by capillary electrophoresis, and the RDI was computed from the resulting electropherogram data using a proprietary algorithm developed by Rna Diagnostics (Sudbury, ON, Canada).

### Cell counting and recovery assays

The effect of chemotherapy agents and other stressors on cell numbers and on cellular replicative capacity in nutrient-rich medium post-treatment was measured as described in our prior publication^25^. Briefly, in the former assay, floating and adherent cells present in cell culture flasks were counted prior to and at various time points after the addition of drug or application of the stressor. In the latter assay, after application of the drug or stressor for a 72-h period, cells were re-seeded in tissue culture plates with drug-free medium. After a 96-h incubation period, the culture confluence was measured using the IncuCyte S3 Live-Cell Analysis System (Sartorius) and normalized to the average culture confluence at 2 h post-seeding.

### Flow cytometry

Following drug treatment, adherent and floating cells were collected as described for the cell counting and recovery assays. All collected cells were then pelleted by centrifugation at 233×*g* for 10 min, resuspended in 3 mL PBS, and pelleted again. The cell pellet was then resuspended in 1 mL PBS. Cell suspensions were placed on ice and fixed by adding 3 mL cold (− 20ºC) anhydrous ethanol (Commercial Alcohols). Fixed cells were stored at − 20ºC until further use. Prior to flow cytometric analysis, fixed cells were stained with propidium iodide. Briefly, fixed cells were harvested by centrifugation at 530×*g* for 5 min, resuspended in 3 mL PBS, and pelleted again. After discarding the supernatant, fixed cells were resuspended in 500 μL propidium iodide staining solution (100 μg mL^-1^ propidium iodide, 100 μg mL^-1^ RNase A, 0.3% NP-40 and 0.1% sodium citrate). Samples were incubated at 37 °C for 30 min prior to flow cytometric analysis. Flow cytometry was performed with the Cytomics FC500 flow cytometer (Beckman Coulter). For each sample, 20,000 events were analyzed via the 675-nm bandpass filter (488-nm excitation wavelength). Data analysis was performed using the CXP Analysis software (Beckman Coulter) without the use of gating.

### Northern blot analysis

Total RNA was isolated from A2780 cells treated with or without 1 μM doxorubicin for 48 h as described in our prior study^28^. The RNA preparations (1 µg per lane) were resolved by denaturing gel electrophoresis using the tricine-triethanolamine method for separating long RNAs, described by Mansour and Pestov^63^. Briefly, RNA preparations were size-separated on 1% agarose gels in a pK_a_-matched buffer comprising 30 mM tricine and 30 mM triethanolamine, with 0.4 M formaldehyde. Following transfer of the resolved RNA preparations to polyvinylidene membranes (Westran S, Sigma-Aldrich) and UV-induced cross-linking of the RNAs, the membranes were blotted with radiolabeled oligodeoxyribonucleotide probes designed to hybridize with the 5′ (28S-7 probe, CTG GCT TCG CCC TGC CCA GGC ATA GTT CAC CAT CTT TCG) or 3′ (28S-5 probe, GAC CCA GAA GCA GGT CGT CTA CGA ATG GTT TAG CGC CAG) end of the 28S rRNA. The DNA probes were synthesized by Integrated DNA Technologies, and then labeled using [γ-^32^P]ATP (Perkin Elmer) and the DNA 5′-end labeling system by Promega. The sequence for the 28S-5 probe was published previously^27^, and the 28S-7 probe was designed using the IDT PrimerQuest tool and the NCBI Primer-Blast tool on the human 28S rRNA (NCBI reference sequence NR_146118.1)^64^. Hybridizations were performed according to Brown and Mackey^65^. Blots were then sealed in bags and exposed to phosphorimaging screens. A Bio-Rad Molecular Imager FX (Bio-Rad Laboratories) was used to scan the exposed screens.

### Statistical analyses

All statistical analyses were carried out using R version 4.0.3^66^ and RStudio version 1.3.1093^67^. To study the effect of various chemotherapy agents and cellular stressors on RNA disruption in the A2780, K562, MDA-MB-231 and A375 cell lines, the RDIs of the treated samples were normalized to that of their cognate untreated control, and one-sample one-tailed *t* tests were carried out to determine if these normalized RDIs were significantly greater than 1, the normalized RDI of the untreated control sample. To this end, the *t test* function from the *stats* package^66^ was used. Normal distribution of the normalized RDIs was confirmed for each data set by graphing normal quantile–quantile plots and conducting Shapiro–Wilk tests using the *shapiro.test* function from the *stats* package^66^. When necessary, normalized RDIs were log_10_-transformed in order to respect the assumption of normality. Data sets that did not meet the requirements of the test were analyzed using non-parametric one-sample one-tailed Wilcoxon signed-rank tests (*wilcox.test* function from the *stats* package^66^).

To determine the effect of doxorubicin, docetaxel and irinotecan concentration on (i) total cell counts, (ii) cell recovery post-treatment, (iii) cellular DNA content and (iv) RNA disruption, fixed-effect one-way ANOVAs were conducted using the *Anova* function from the *car* package^68^. Adherence to the test’s assumptions was confirmed for each data set, as follows. The assumption of normality was tested by plotting normal quantile–quantile plots of the models’ residuals, and by conducting Shapiro–Wilk tests on the models’ residuals using the *shapiro.test* function from the *stats* package^66^. The assumption of homoscedasticity was tested by plotting the models’ fitted values against their residuals, and by performing Bartlett’s tests using the *bartlett.test* function from the *stats* package^60^. When necessary, the collected data were log_10_- or power-transformed in order to respect these assumptions. When data sets respected the assumption of normality, but not the assumption of homoscedasticity, Welch’s ANOVAs were conducted using the *oneway.test* function from the *stats* package^66^. When data sets did not respect the assumption of normality, non-parametric Kruskal–Wallis rank-sum tests were carried out using the *kruskal.test* function from the *stats* package^66^. Post hoc multiple comparisons to the ‘0 h’ (total cell counts) or untreated (cell recovery, cellular DNA content and RNA disruption) control were then carried out using either one-tailed Dunnett’s tests (following ANOVAs), Tamhane-Dunnett tests (following Welch’s ANOVAs) or Conover’s tests (following Kruskal–Wallis rank-sum tests), as implemented in the *dunnettTest*, *tamhaneDunnettTest* and *kwManyOneConoverTest* functions from the *PMCMRplus* package^69^, respectively.

The impact of doxorubicin concentration and exposure length on RNA disruption in the A2780, K562, MDA-MB-231 and A375 cell lines was investigated by performing fixed-effect type III two-way ANOVAs using the *Anova* function from the *car* package^68^. Adherence to the test’s assumptions was confirmed for each data set, as described for one-way ANOVAs. When necessary, the collected data were log_10_- or rank-transformed in order to conform to the assumptions. Post hoc multiple comparisons of estimated marginal means were conducted using the *emmeans* function from the *emmeans* package^70^. *P* values were adjusted for multiple comparisons using the Tukey method.

*P* values equal to, or less than, 0.05 were deemed statistically significant. Unless otherwise stated, raw untransformed data were plotted.

### Graphics

Electropherograms and flow cytometry plots/histograms were produced by the 2100 Expert software version B.02.09.SI725 (SR1) (Agilent Technologies) and the CXP Analysis software version 2.2 (Beckman Coulter), respectively. Plots were prepared using R version 4.0.3^66^ with RStudio version 1.3.1093^67^.

## Supplementary Information

Supplementary Information.

## Data Availability

The details of all statistical tests performed for the data depicted in Figs. 2, 3, 4 and 5, the images of all RNA electropherograms, the flow cytometric DNA content plots and histograms, and the data from *Mycoplasma* experiments can all be found in the supplementary information file. The full RNA electropherogram peak quantification datasets used and/or analyzed during the current study are available from the corresponding author on reasonable request.
