# Supplementary material for: RNA disruption is a widespread phenomenon associated with stress-induced cell death in tumour cells
Source: Sci Rep. 2023 Jan 31;13:1711. doi: 10.1038/s41598-023-28635-8 (PMC9889758; doi:10.1038/s41598-023-28635-8)
Supplement: Supplementary file 1 — Supplementary Information. [file 41598_2023_28635_MOESM1_ESM.pdf]

Scientific Reports

Supplementary Information

RNA disruption is a widespread phenomenon associated with stress-induced cell death in tumour cells

Phillipe Butler[1], Isabella Pascheto[1], Michayla Lizzi[1], Renée St-Onge[2], Carita Lanner[1, 4], Baoqing Guo[3], Twinkle Masilamani[2], Laura B. Pritzker[2], A. Thomas Kovala[1, 4], and Amadeo M. Parissenti[1, 2, 3, 4] *

[1] Graduate Program in Chemical Sciences, Laurentian University, Sudbury, ON, Canada

[2] Rna Diagnostics, Inc., Sudbury and Toronto, ON, Canada

[3] Health Sciences North Research Institute, Sudbury, ON, Canada

[4] Division of Medical Sciences, Northern Ontario School of Medicine, Sudbury, ON, Canada

Corresponding author: Amadeo M. Parissenti, telephone: 1-705-522-6237 ext. 2719, e-mail: aparissenti@hsnri.ca

Supplementary Table S1. Results of statistical analyses of the data shown in Fig. 2.

A2780
DOX: One-sample t-test, $t(9) = 21.66$, $n = 10$, $P < 0.01$
EPI: One-sample t-test, $t(3) = 4.21$, $n = 4$, $P = 0.01$
ETOP: One-sample t-test, $t(4) = 11.09$, $n = 5$, $P < 0.01$
CBN: One-sample t-test, $t(3) = 2.47$, $n = 4$, $P = 0.05$
CIS: One-sample t-test, $t(5) = 3.38$, $n = 6$, $P < 0.01$
TAX: One-sample t-test, $t(4) = 2.53$, $n = 5$, $P = 0.03$
DXL: One-sample t-test, $t(3) = 4.90$, $n = 4$, $P < 0.01$
VIN: One-sample t-test, $t(4) = 5.46$, $n = 5$, $P < 0.01$
IRN: One-sample t-test, $t(3) = 2.93$, $n = 4$, $P = 0.03$
PBC: One-sample Wilcoxon signed-rank test, V = 3, $n = 2$, $P = 0.25$

K562
DOX: One-sample t-test, $t(5) = 11.34$, $n = 6$, $P < 0.01$
EPI: One-sample t-test, $t(2) = 5.43$, $n = 3$, $P = 0.02$
ETOP: One-sample t-test, $t(2) = 2.06$, $n = 3$, $P = 0.09$
CBN: One-sample Wilcoxon signed-rank test, V = 3, $n = 3$, $P = 0.17$
CIS: One-sample Wilcoxon signed-rank test, V = 1, $n = 3$, $P = 0.50$
TAX: One-sample t-test, $t(2) = 1.78$, $n = 3$, $P = 0.11$
DXL: One-sample t-test, $t(2) = 2.65$, $n = 3$, $P = 0.06$
VIN: One-sample t-test, $t(2) = 1.96$, $n = 3$, $P = 0.09$
IRN: One-sample t-test, $t(2) = 1.73$, $n = 3$, $P = 0.11$
PBC: One-sample Wilcoxon signed-rank test, V = 6, $n = 3$, $P = 0.09$

MDA-MB-231
DOX: One-sample t-test, $t(5) = 3.73$, $n = 6$, $P < 0.01$
EPI: One-sample t-test, $t(2) = 2.75$, $n = 3$, $P = 0.06$
ETOP: One-sample t-test, $t(2) = 6.50$, $n = 3$, $P = 0.01$
CBN: One-sample Wilcoxon signed-rank test, V = 3, $n = 3$, $P = 0.17$
CIS: One-sample t-test, $t(2) = 1.00$, $n = 3$, $P = 0.21$
TAX: One-sample Wilcoxon signed-rank test, V = 6, $n = 3$, $P = 0.09$
DXL: One-sample t-test, $t(2) = 2.10$, $n = 3$, $P = 0.09$
VIN: One-sample t-test, $t(2) = 2.20$, $n = 3$, $P = 0.08$
IRN: One-sample t-test, $t(2) = 1.39$, $n = 3$, $P = 0.15$
PBC: One-sample Wilcoxon signed-rank test, V = 3, $n = 2$, $P = 0.25$

A375
DOX: One-sample t-test, $t(6) = 5.67$, $n = 7$, $P < 0.01$
EPI: One-sample t-test, $t(3) = 2.91$, $n = 4$, $P = 0.03$
ETOP: One-sample t-test, $t(3) = 6.80$, $n = 4$, $P < 0.01$
CBN: One-sample t-test, $t(3) = 2.81$, $n = 4$, $P = 0.03$
CIS: One-sample t-test, $t(3) = 1.90$, $n = 4$, $P = 0.08$
TAX: One-sample t-test, $t(3) = 2.82$, $n = 4$, $P = 0.03$
DXL: One-sample t-test, $t(3) = 3.64$, $n = 4$, $P = 0.02$
VIN: One-sample t-test, $t(3) = 1.78$, $n = 4$, $P = 0.09$
IRN: One-sample t-test, $t(3) = 3.64$, $n = 4$, $P = 0.02$
PBC: One-sample t-test, $t(2) = 3.12$, $n = 3$, $P = 0.04$

Supplementary Table S2. Results of statistical analyses of the data shown in Fig. 3.

A2780
TPG: One-sample *t*-test, $t(2) = 16.52$, $n = 3$, $P < 0.01$
TUN: One-sample Wilcoxon signed-rank test, V = 6, $n = 3$, $P = 0.09$
CHX: One-sample Wilcoxon signed-rank test, V = 10, $n = 4$, $P = 0.05$
Medium: One-sample *t*-test, $t(2) = 1.65$, $n = 3$, $P = 0.12$
H2O2: One-sample *t*-test, $t(3) = 5.83$, $n = 4$, $P < 0.01$

K562
TPG: One-sample *t*-test, $t(2) = 8.71$, $n = 3$, $P < 0.01$
TUN: One-sample Wilcoxon signed-rank test, V = 3, $n = 3$, $P = 0.17$
CHX: One-sample *t*-test, $t(2) = 4.62$, $n = 3$, $P = 0.02$
Medium: One-sample *t*-test, $t(2) = 2.43$, $n = 3$, $P = 0.07$
H2O2: One-sample *t*-test, $t(2) = 3.90$, $n = 3$, $P = 0.03$

MDA-MB-231
TPG: One-sample Wilcoxon signed-rank test, V = 3, $n = 2$, $P = 0.25$
TUN: One-sample Wilcoxon signed-rank test, V = 6, $n = 3$, $P = 0.09$
CHX: One-sample *t*-test, $t(2) = 3.12$, $n = 3$, $P = 0.04$
Medium: One-sample *t*-test, $t(2) = 4.15$, $n = 3$, $P = 0.03$
H2O2: One-sample *t*-test, $t(4) = 3.15$, $n = 5$, $P = 0.02$

A375
TPG: One-sample *t*-test, $t(3) = 4.15$, $n = 4$, $P = 0.01$
TUN: One-sample *t*-test, $t(3) = 3.78$, $n = 4$, $P = 0.02$
CHX: One-sample *t*-test, $t(3) = 3.42$, $n = 4$, $P = 0.02$
Medium: One-sample *t*-test, $t(3) = 3.62$, $n = 4$, $P = 0.02$
H2O2: One-sample *t*-test, $t(2) = 5.86$, $n = 3$, $P = 0.01$

Supplementary Table S3. Results of statistical analyses of the data shown in Fig. 4.

RNA disruption assay
Doxorubicin: One-way ANOVA, $F(10, 22) = 12.46$, $n = 3$, $P < 0.01$
Docetaxel: One-way ANOVA, $F(11, 24) = 7.31$, $n = 3$, $P < 0.01$
Irinotecan: Kruskal-Wallis rank-sum test, $\chi^2(11) = 44.85$, $n = 6$, $P < 0.01$

Cell counting assay
Doxorubicin: One-way ANOVA, $F(12, 26) = 131.17$, $n = 3$, $P < 0.01$
Docetaxel: One-way ANOVA, $F(12, 26) = 153.91$, $n = 3$, $P < 0.01$
Irinotecan: One-way ANOVA, $F(12, 26) = 54.08$, $n = 3$, $P < 0.01$

Recovery assay
Doxorubicin: One-way ANOVA, $F(11, 24) = 191.37$, $n = 3$, $P < 0.01$
Docetaxel: One-way ANOVA, $F(11, 24) = 92.24$, $n = 3$, $P < 0.01$
Irinotecan: One-way ANOVA, $F(11, 24) = 108.54$, $n = 3$, $P < 0.01$

DNA content analysis
Doxorubicin: One-way ANOVA, $F(11, 24) = 78.02$, $n = 3$, $P < 0.01$
Docetaxel: One-way ANOVA, $F(11, 24) = 72.46$, $n = 3$, $P < 0.01$
Irinotecan: One-way ANOVA, $F(11, 24) = 40.08$, $n = 3$, $P < 0.01$

Supplementary Table S4. Results of statistical analyses of the data shown in Fig. 5.

RNA disruption assay
HUVEC: One-way ANOVA, $F(5, 16) = 8.62$, $n = 2\text{-}4$, $P < 0.01$
NIH3T3: Kruskal-Wallis rank-sum test, χ^2 (5) = 3.44, $n = 4$, $P = 0.63$
MCF-10A: Kruskal-Wallis rank-sum test, χ^2 (5) = 11.51, $n = 4$, $P = 0.04$

Cell counting assay
HUVEC: Welch's ANOVA, $F(6.00, 8.24) = 59.4$, $n = 4$, $P < 0.01$
NIH3T3: One-way ANOVA, $F(6, 21) = 299.8$, $n = 4$, $P < 0.01$
MCF-10A: One-way ANOVA, $F(6, 21) = 261.16$, $n = 4$, $P < 0.01$

DNA content analysis
HUVEC: One-way ANOVA, $F(5, 18) = 155.25$, $n = 4$, $P < 0.01$
NIH3T3: One-way ANOVA, $F(5, 18) = 28.64$, $n = 4$, $P < 0.01$
MCF-10A: Welch's ANOVA, $F(5.00, 7.13) = 48.45$, $n = 4$, $P < 0.01$

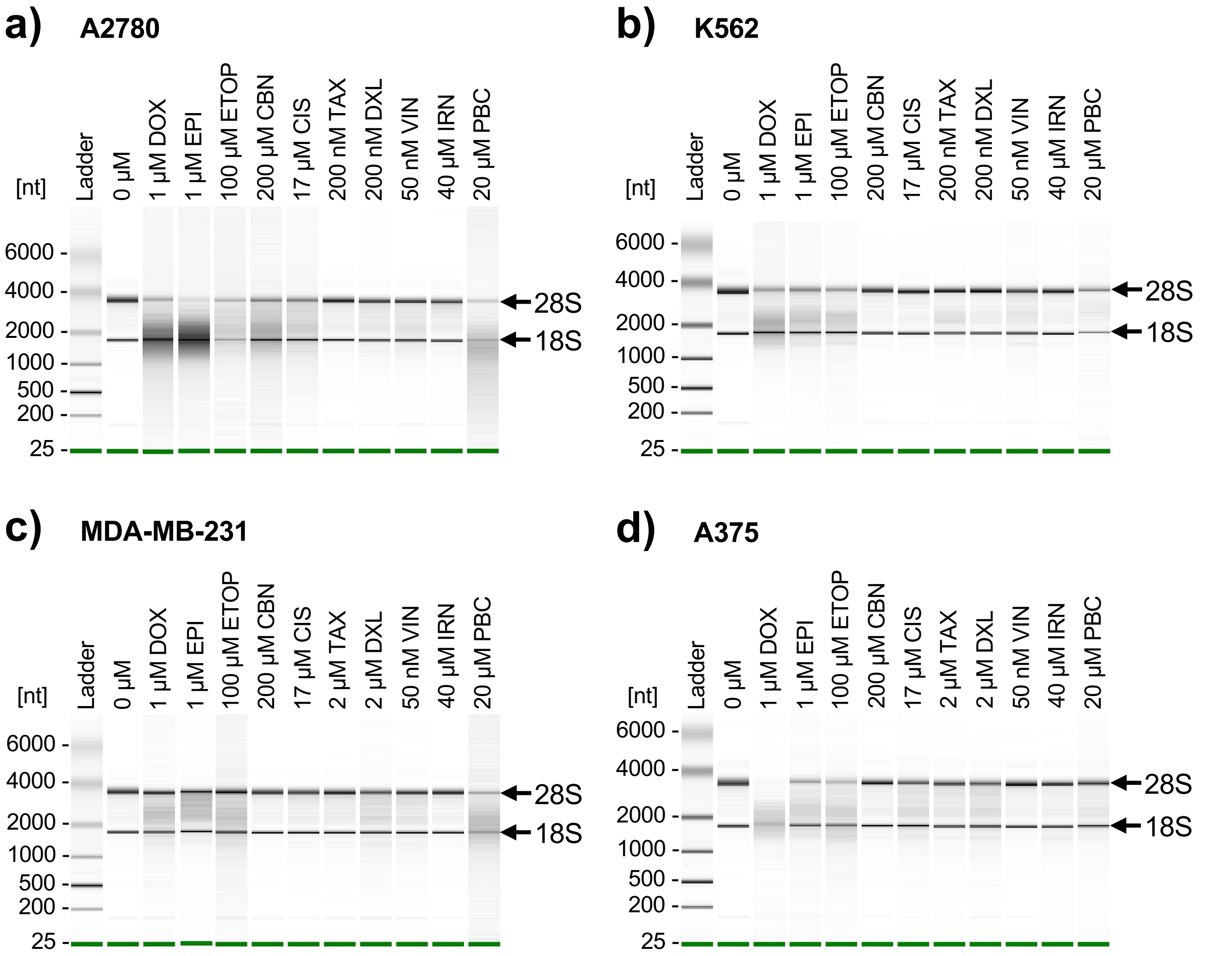

Supplementary Figure S1. RNA disruption induced by multiple chemotherapy agents in different cell lines. A2780 **(a)**, K562 **(b)**, MDA-MB-231 **(c)** and A375 **(d)** cells were exposed to various chemotherapy agents for 72 h. Total RNA was isolated from cells following drug treatment, and size-separated by capillary gel electrophoresis. Arrows indicate the position of the full-length 28S and 18S rRNA bands. Each electropherogram is representative of at least three independent biological replicates. DOX, doxorubicin; EPI, epirubicin; ETOP, etoposide; CBN, carboplatin; CIS, cisplatin; TAX, paclitaxel; DXL, docetaxel; VIN, vincristine; IRN, irinotecan; PBC, palbociclib.

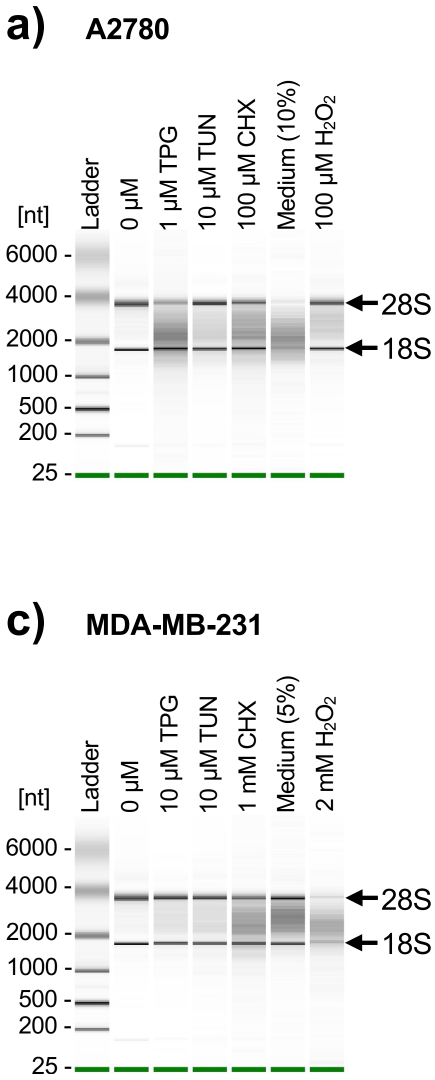

Supplementary Figure S2. RNA disruption induced by various cellular stressors in multiple cell lines. A2780 **(a)**, K562 **(b)**, MDA-MB-231 **(c)** and A375 **(d)** cells were exposed to various cellular stressors for 72 h. Total RNA was isolated from cells following drug treatment, and size-separated by capillary gel electrophoresis. Arrows indicate the position of the full-length 28S and 18S rRNA bands. Each electropherogram is representative of at least three independent biological replicates. TPG, thapsigargin; TUN, tunicamycin; CHX, cycloheximide; Medium, standard culture medium diluted to 5 or 10% in phosphate-buffered saline.

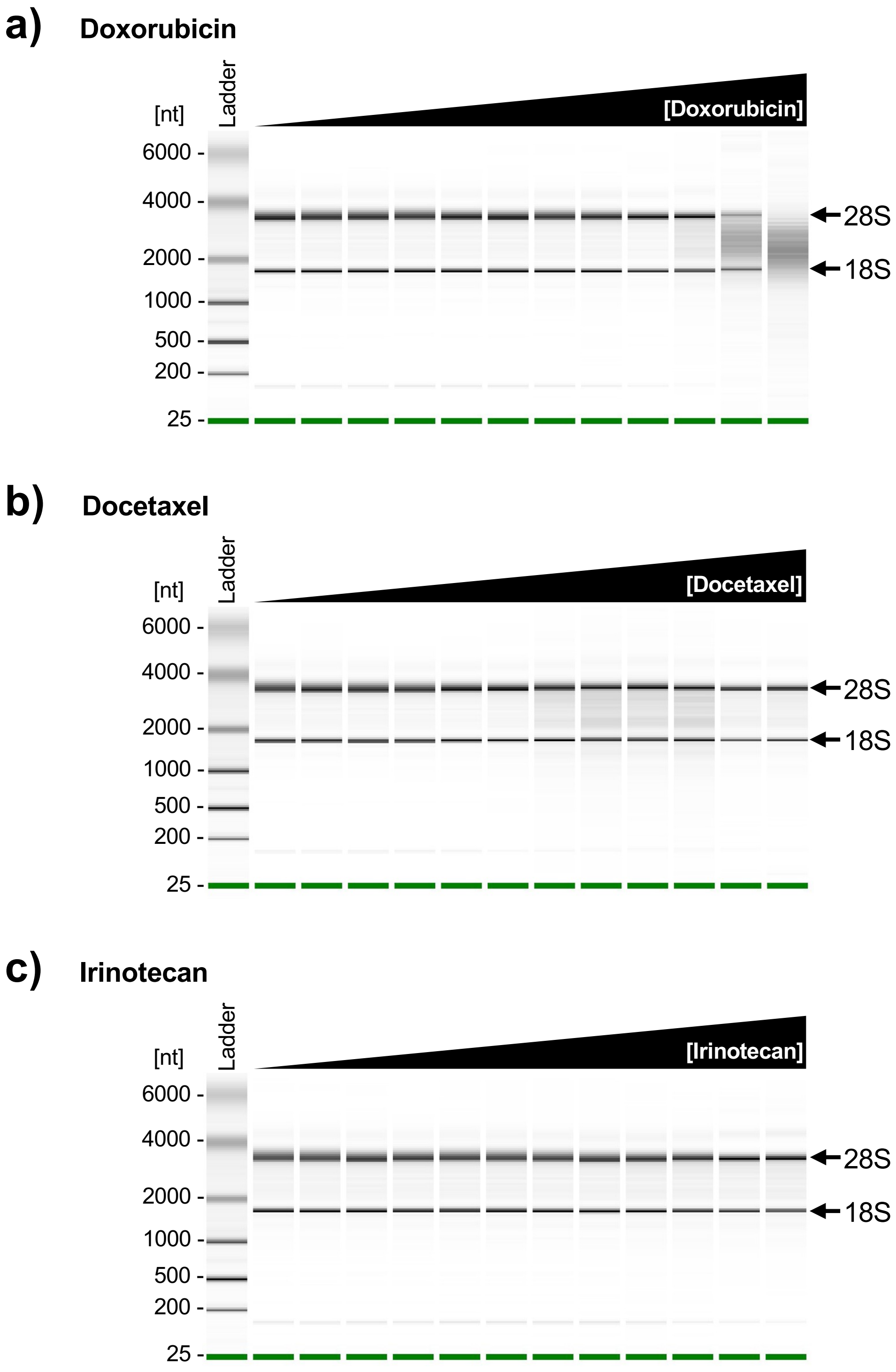

Supplementary Figure S3. Effect of three chemotherapy agents on RNA integrity. A2780 cells were treated with different concentrations of doxorubicin **(a)**, docetaxel **(b)** or irinotecan **(c)** for 72 h. Total RNA was isolated from cells, and size-separated by capillary gel electrophoresis. Arrows indicate the position of the full-length 28S and 18S rRNA bands. Each electropherogram is representative of at least three independent biological replicates.

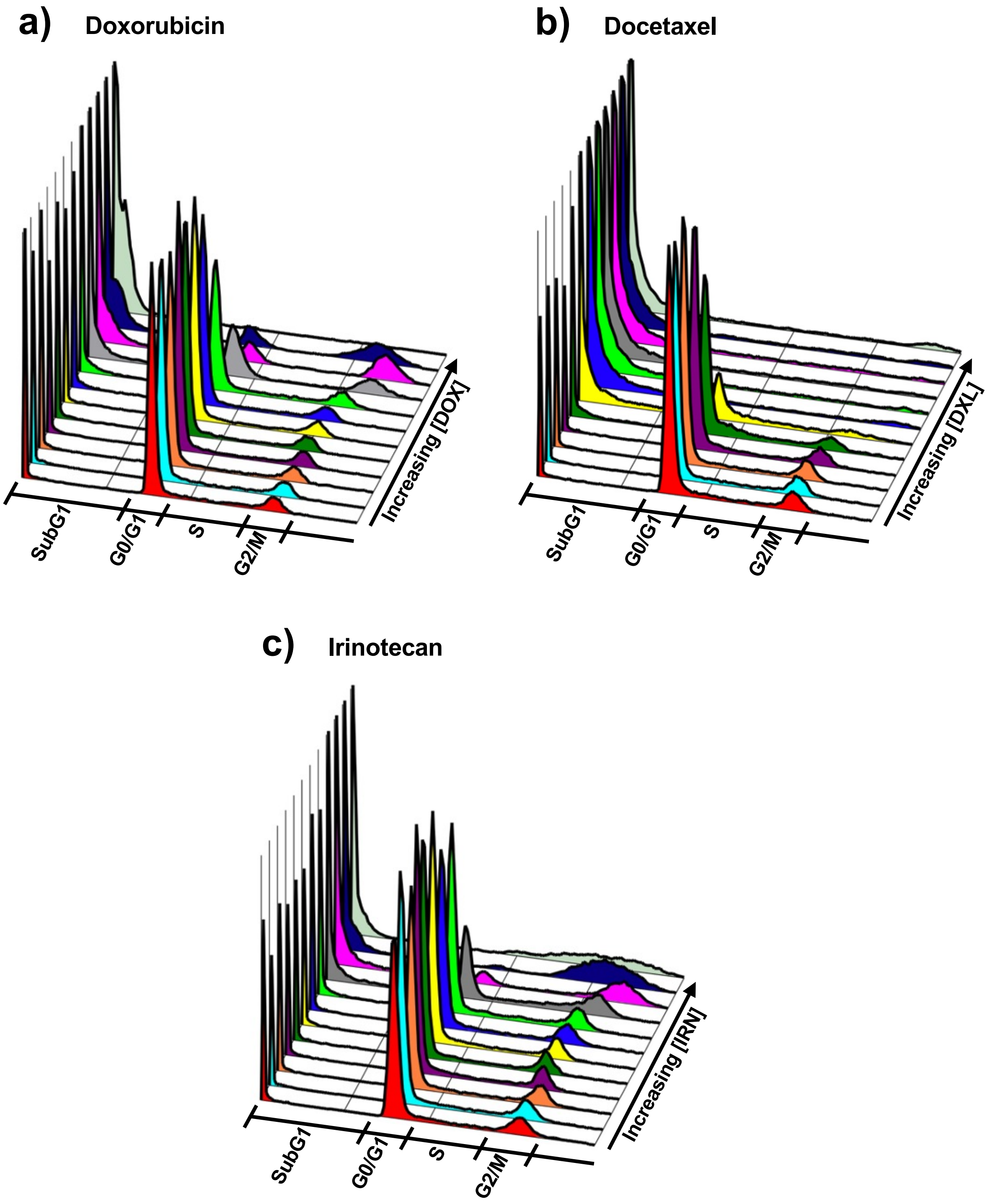

Supplementary Figure S4. Effect of three chemotherapy agents on cellular DNA content. A2780 cells were treated with different concentrations of doxorubicin **(a)**, docetaxel **(b)** or irinotecan **(c)** for 72 h. Drug-treated cells were collected, washed, fixed, stained with propidium iodide, and then analyzed by flow cytometry. Cell cycle stages are specified under each histogram. Each histogram is representative of at least three independent biological replicates. DOX, doxorubicin; DXL, docetaxel; IRN, irinotecan.

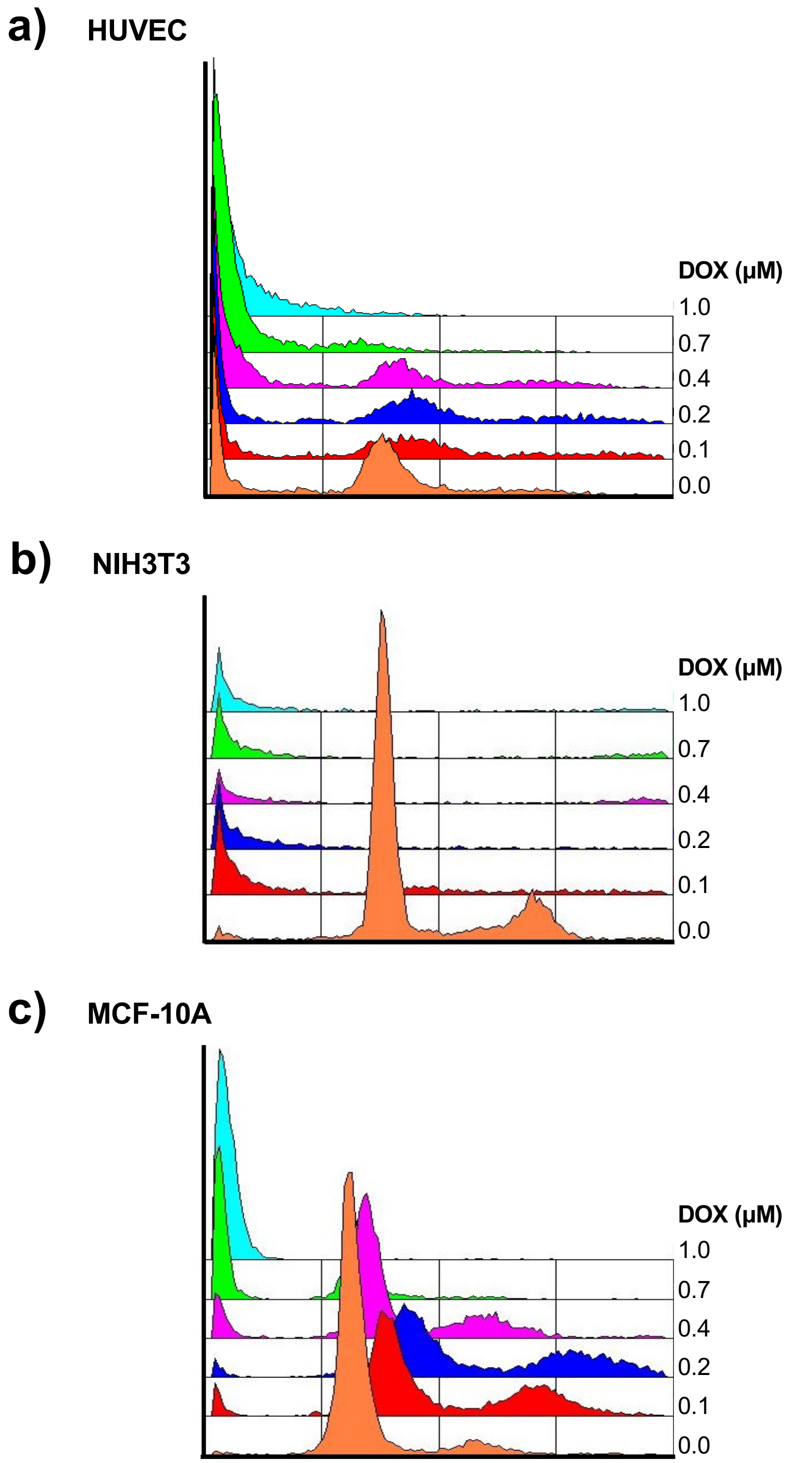

Supplementary Figure S5. Effect of doxorubicin on the cellular DNA content of three non-tumorigenic cell lines. HUVEC **(a)**, NIH3T3 **(b)** and MCF-10A **(c)** cells were treated with different concentrations of doxorubicin for 72 h. Drug-treated cells were collected, washed, fixed, stained with propidium iodide, and then analyzed by flow cytometry. Each histogram is representative of at least four independent biological replicates. DOX, doxorubicin.

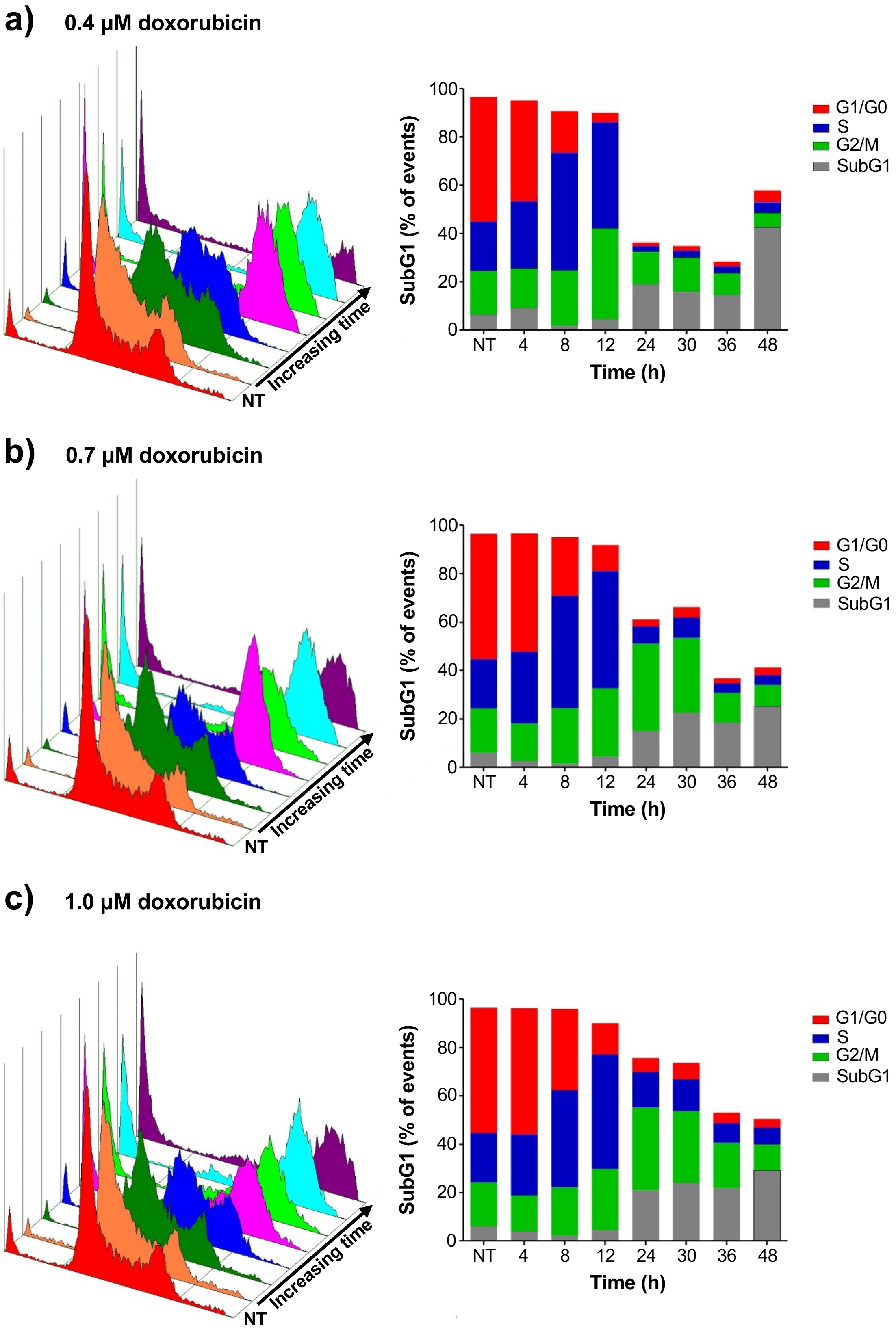

Supplementary Figure S6. Effect of doxorubicin on the cellular DNA content of NIH3T3 cells. Cells were left untreated for 48 h (NT) or treated with 0.4 µM **(a)**, 0.7 µM **(b)** or 1.0 µM **(c)** doxorubicin for 4 to 48 h. Cells were collected, washed, fixed, stained with propidium iodide, and then analyzed by flow cytometry.

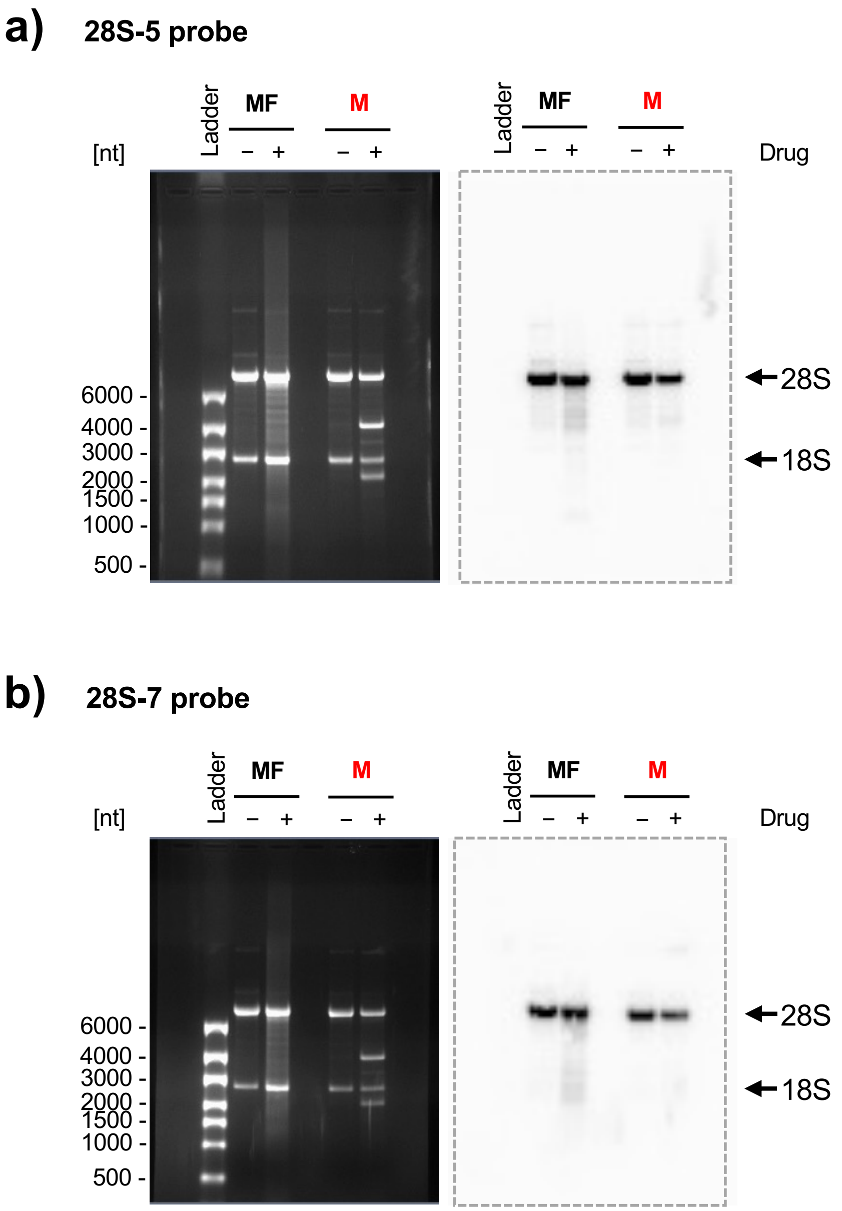

Supplementary Figure S7. Origin of RNA disruption products. Total RNA was isolated from *Mycoplasma*-free (MF) and *Mycoplasma*-infected (M) A2780 cells treated with or without 1 μM doxorubicin (*Mycoplasma*-free cells) or 200 nM docetaxel (infected cells). The RNA was then resolved by denaturing gel electrophoresis and transferred onto a PVDF membrane prior to hybridization with radiolabeled DNA probes 28S-5 (**a**) or 28S-7 (**b**). UV-visualized, ethidium bromide-stained agarose gel images (left panels) and autoradiograms of the northern blots (right panels) are shown for the respective 28S rRNA probes. Arrows indicate the position of the full-length 28S and 18S rRNA bands. Gray hatched boxes indicate the limits of the blot. Each gel image and autoradiogram are representative of two independent biological replicates.

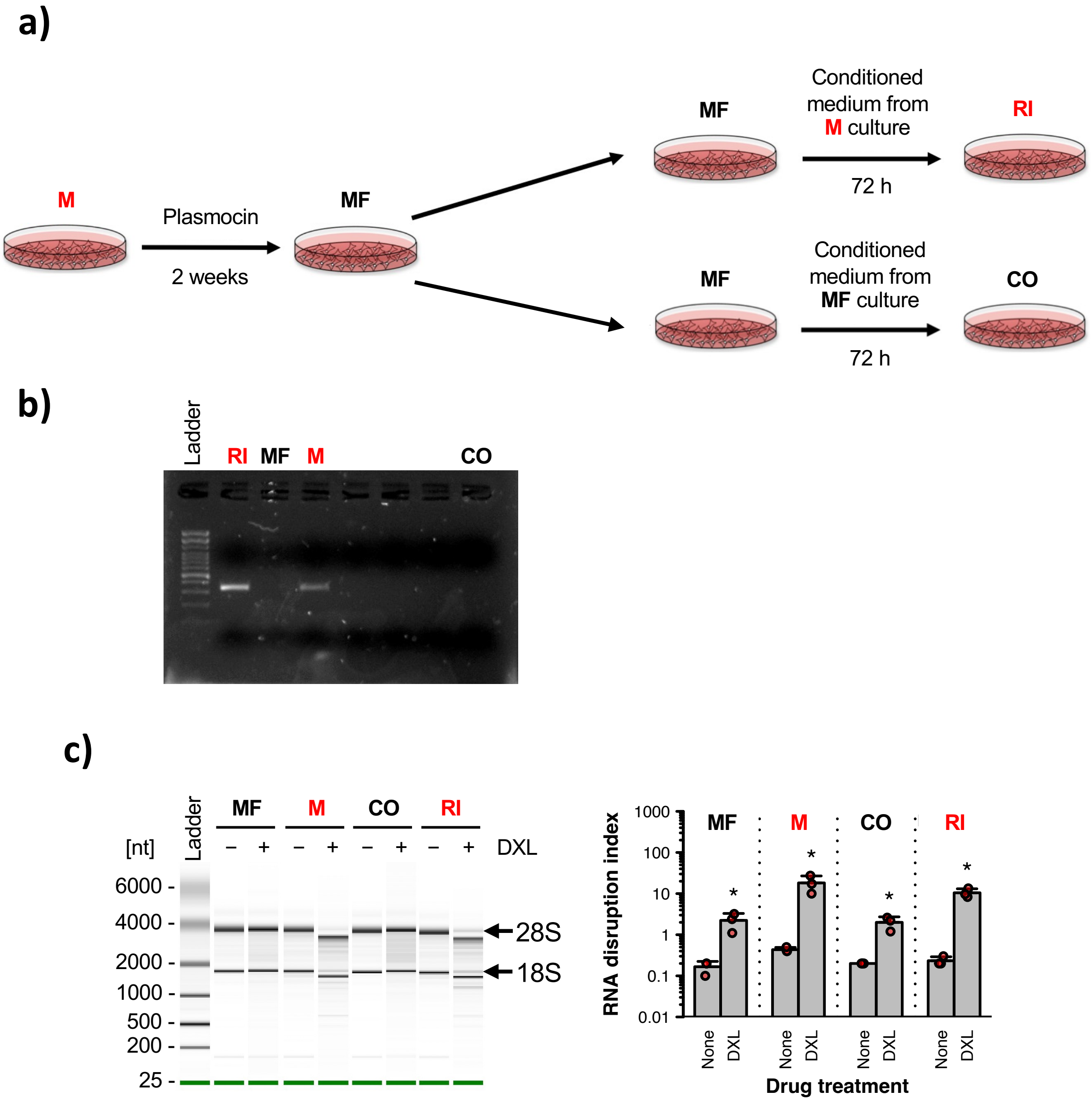

Supplementary Figure S8. Impact of *Mycoplasma* infection on chemotherapy-induced RNA disruption. **(a)** Experimental design. *Mycoplasma*-infected A2780 cells (M) were treated with plasmocin for 2 weeks to generate *Mycoplasma*-free A2780 cells (MF). *Mycoplasma*-free cells were subsequently cultivated for 72 h in conditioned, cell-free medium derived from a *Mycoplasma*-infected culture to generate re-infected A2780 cells (RI). As a control, cells were also cultivated for 72 h in conditioned, cell-free medium derived from a *Mycoplasma*-free culture (CO). Black labels, *Mycoplasma*-free cells; red labels, *Mycoplasma*-infected cells. **(b)** *Mycoplasma* contamination status. Presence or absence of *Mycoplasma* in the four cell lines was assessed by PCR. **(c)** RNA disruption assay. Cells were treated with (+) or without (-) docetaxel (DXL) for 72 h. Total RNA was isolated from cells following drug treatment, and RNA disruption was analyzed using the RDA. ***Left panel.*** Virtual gel image of isolated RNA. Arrows indicate the position of the full-length 28S and 18S rRNA bands. The electropherogram is representative of three independent biological replicates. ***Right panel.*** RNA disruption quantified using the RDA. Data are presented as means ± standard deviation, with individual data points shown in red. A two-way ANOVA revealed a significant interaction between drug treatment and the *Mycoplasma* infection status [$F(3, 16) = 7.19$, $n = 3$, $P < 0.01$]. For a given *Mycoplasma* infection status, treatment groups labelled with an asterisk are significantly different from the untreated control.
